# Circ_0020460 drives tumorigenesis in cervical cancer through miR-485-3p sponging

**DOI:** 10.1007/s12672-024-00933-1

**Published:** 2024-03-27

**Authors:** Kun Yan, Chunyan Hu, Yali Cheng, Lingzhi Zheng, Baojin Zeng, Sujun Zhao, Chen Liu

**Affiliations:** 1 Department of Gynecology, Northwest Women’s and Children’s Hospital, Xi’an, 710016 China; 2 Department of Gynecology, Taizhou Hospital of Zhejiang Province Affiliated to Wenzhou Medical University, Enze Hospital, Taizhou Enze Medical Center (Group), No. 1, Tongyang Road, Luqiao District, Taizhou, 318050 China

**Keywords:** Circ_0020460, miR-485-3p, CXCL1, Cervical cancer

## Abstract

Deregulation of circular RNAs (circRNAs) is widely recognized in cancer progression. Our study aims to investigate the role of circ_0020460 in the development of cervical cancer (CC) and its potential mechanism of action. Quantitative real-time polymerase chain reaction (qRT-PCR) and western blot assays were used to detect the expression levels of circ_0020460, miR-485-3p and C-X-C motif chemokine ligand 1 (CXCL1). The roles of circ_0020460 on cell proliferation, cell migration, cell invasion, cell apoptosis, and angiogenesis were investigated using cell counting kit-8 (CCK-8) and Ethynyl deoxyuridine (Edu) assay, wound healing assay, transwell assay, flow cytometry assay, and tube formation assay, respectively. The putative relationship predicted by bioinformatics analysis was verified by dual-luciferase reporter assay and RNA immunoprecipitation (RIP) assay. Xenograft models were constructed to explore the role of circ_0020460 in vivo. The expression of circ_0020460 and CXCL1 expression were increased, while miR-485-3p expression was declined in CC tissues and cells. Circ_0020460 knockdown suppressed CC cell proliferation, cell migration, cell invasion, angiogenesis, and promoted cell apoptosis. Circ_0020460 functioned as a miR-485-3p sponge to inhibit miR-485-3p level, and the anti-cancer effects mediated by circ_0020460 knockdown were reversed by miR-485-3p inhibitor. MiR-485-3p bound to CXCL1 3ʹ untranslated region (3ʹUTR) to degrade CXCL1 expression, and the anti-cancer effects of miR-485-3p restoration were impaired by CXCL1 overexpression. Circ_0020460 downregulation inhibited CC xenograft tumor growth. These results suggest that circ_0020460 promoted the malignant behavior of CC cells by modulating the miR-485-3p/CXCL1 axis.

**Supplementary Information:**

The online version contains supplementary material available at 10.1007/s12672-024-00933-1.

## Introduction

Cervical cancer is a common in the world [1]. With the improvement of economic level, the enhancement of women’s awareness of health care and the wide application of cervical cancer screening technology, more and more cervical cancer patients are found in the early stage, increasing the chances of patients being cured by surgery [2, 3]. However, the high infection rate of HPV (human papillomavirus) is accompanied by high incidence of cervical cancer, which not only put heavy financial pressure on cancer prevention and screening, but also suggests that there are important factors such as inter-individual genetic differences in cancer development and progression [4–6]. Therefore, it is very important to explore the exact pathogenesis of CC and to find new potential therapeutic targets.

There is only less than 2% nucleic acid sequences that can encode proteins in the human genome, actually, most of the genes are transcribed into non-coding RNAs [7]. In recent years, circRNAs (circular RNAs), as another important non-coding RNAs after microRNAs and lncRNAs, have attracted considerable attention [8, 9]. CircRNAs were first discovered by Sanger et al*.* in plant viroids and Sendai virus successively in 1976 [10, 11]. Subsequently, Hus et al*.* clearly observed the circular structure of circRNAs in the cytoplasm under electron microscopy in 1979 [12]. In the 1990s, Nigro et al*.* revealed that in addition to splicing the exons together in a certain order to form a mature linear mRNA, there is also a special phenomenon of reverse splicing, i.e., the formation of reverse cyclization between downstream exons and upstream exons, and finally a single-stranded connected closed-loop structure is formed [13]. It has been found that circRNAs could be used as competitive endogenous RNA to regulate target gene expression [14].

At present, more and more differential expression circRNAs between cancer tissues and normal tissues were found by using sequencing technology. Hsa_circ_002059 was highly correlated with distal and lymph node metastasis of gastric cancer [15]. Through chip screening, it was found that hsa_circ_100855 was dramatically up-regulated and hsa_circ_104912 was significantly down-regulated in laryngeal cancer tissues, and the expression levels of hsa_circ_100855 and hsa_circ_104912 were correlated with tumor stage, differentiation and lymphatic metastasis, and were expected to become tumor markers for laryngeal cance [16]. Zu et al*.* confirmed that circDOCK1 (hsa_circ_0020394), as a marker of bladder carcinoma, was upregulated in bladder carcinoma, and it could inhibit miR-132-3p and promote tumor growth in vivo and in vitro [17]. Zhang et al*.* reported that circ_DOCK1 (hsa_circ_0020397) was significantly upregulated in colorectal cancer tissues, and it was significantly correlated with the progression of colon cancer cells [18]. DOCK1 is a host gene corresponding to multiple circRNA IDs, and circ_0020460 was one of them. Circ_0020460 is derived from exon 22–23 of DOCK1 (dedicator of cytokinesis 1, chr10:128850944–128860040) with a mature length of 244 bp. Circ_0020460 was known to be a highly expressed circRNA in intracranial aneurysms [19], but the functional studies of circ_0020460 have not been reported. The specific expression and mechanism of circ_DOCK1 in colorectal cancer and bladder cancer have been reported, but there is no literature regarding the effect of circ_DOCK1 in cervical cancer. In this study, through the analysis of public database, the differentially expressed circRNAs were selected in cervical cancer tissues and corresponding normal tissues. qPCR experiments were used to analyze the expression level of circ_0020460 in CC tissues and corresponding normal tissues. The function of circ_0020460 and possible pathogenesis in cervical cancer were revealed. Our study might provide a new direction for molecular targeted therapy of cervical cancer.

## Materials and methods

### Clinical samples and public datasets

53 cervical cancer (CC) patients at Northwest Women’s and Children’s Hospital were enrolled for the study. The normal and cancer tissue samples were acquired from these patients with the approval of the Ethics Committee of Northwest Women’s and Children’s Hospital. It was carried out according to the guidelines of Declaration of Helsinki and in compliance with the ARRIVE guidelines, and all patients included in this study signed the informed consents. All excised tissues were exposed to liquid nitrogen and stored at −80 °C. The basic clinicopathologic information was added in Table 1.

**Table 1:** The clinical details of 53 CC patients

Clinicopathologic features	n	%
Age (years)		
≥ 50	40	75.5
< 50	13	24
Tumor differentiation		
Well + moderate	36	67.9
Poor	17	32.1
Tumor size (cm)		
≥ 3	20	37.7
< 3	33	62.3
TNM stage		
I + II	34	64.2
III	19	35.8
HPV		
+	30	56.6
−	23	43.4

GSE102686 (https://www.ncbi.nlm.nih.gov/geo/query/acc.cgi?acc=GSE102686) was obtained from the GEO dataset, and this dataset provided the sequencing data of circRNAs from 5 cervical squamous cell carcinoma tissues and 5 paired-paracancerous cervical tissues. GSE63678 (https://www.ncbi.nlm.nih.gov/geo/query/acc.cgi?acc=GSE63678) dataset provided the sequencing data for mRNAs of 5 cervical cancer tissues and 5 normal tissues. The online software GEO2R was used to perform differential expression analysis for GSE102686 and GSE63678 datasets.

### RNA extraction and quantitative real-time polymerase chain reaction (qRT-PCR)

Total RNA was extracted using TriQuick Reagent (Solarbio, Beijing, China). The RNAs were quantified using an UV-3100PC spectrophotometer (Agilent, West Lothian, UK) prior to reverse transcription performed with cDNA Synthesis Master Mix (Sangon Biotech, Shanghai, China) and miRNA First Strand cDNA Synthesis (Sangon Biotech). qRT-PCR was performed on Mx3000P system with 1 × SYBR Abstart Master Mix (Sangon Biotech) and PCR forward (0.2 μM) and reverse primers (0.2 μM) (listed in Table 2). The parameters of PCR reaction were set according to the instruction of SYBR Green reagent (TaKaRa, Dalian, China). All data were normalized using the 2^−ΔΔCt^ method with β-actin and U6 as the internal reference for circRNA and mRNA or miRNA.

**Table 2:** Primers sequences used for PCR

Name		Primers for PCR (5ʹ-3ʹ)
circ_0020460	Forward	GAGGCTGACTTCGTGGAATC
	Reverse	TCTAGCGCTTTCATGGCTTT
DOCK1	Forward	CCTAGACGCGGAGTTTCCTG
	Reverse	CCGCTCCTCTGGCATCATAG
CXCL1	Forward	CTGGCTTAGAACAAAGGGGCT
	Reverse	TAAAGGTAGCCCTTGTTTCCCC
miR-485-3p	Forward	GTATGAGTCATACACGGCTCTC
	Reverse	CTCAACTGGTGTCGTGGAG
β-actin	Forward	GACTCCAAGGCCACGGATAG
	Reverse	TGTTCGAGGATCTGTGCCAA
U6	Forward	CTCGCTTCGGCAGCACA
	Reverse	AACGCTTCACGAATTTGCGT

### Cell culture

The ectocervical Ect1/E6E7 cell and CC cell lines (HeLa and CaSki) were obtained from ATCC (Manassas, VA, USA). HeLa cells were cultured in Eagle's Minimum Essential Medium (EMEM; GIBCO, Thermo Fisher Scientific, Rockville, MD, USA) containing 10% fetal bovine serum (Sigma-Aldrich, St. Louis, MO, USA) at 37 °C with 5% CO_2_. CaSki cells were maintained in 90% Roswell Park Memorial Institute 1640 Medium (RPMI-1640; Sigma-Aldrich) with 10% fetal bovine serum (Sigma-Aldrich). The Ect1/E6E7 cells placed in Keratinocyte-Serum Free medium (GIBCO) with bovine pituitary extract (0.05 mg/ml) (GIBCO), human recombinant epidermal growth factor (0.1 ng/ml) (GIBCO), and additional calcium chloride (44.1 mg/l) (GIBCO). HUVECs (human umbilical vein endothelial cells) were obtained from ATCC and cells need to be cultured with complete medium (Thermo Fisher Scientific). Cell passage was performed when cells reached about 90% confluence. All cell lines were grown in 37 °C environment with 5% CO_2_.

### RNase R digestion

To certify the circular characteristics of circ_0020460, the total RNA extracted from HeLa and CaSki cells were incubated with RNase R (Epicenter, Stockholm, Sweden) at 37 °C for 30 min. Later, levels of DOCK1 mRNA and circ_0020460 were assessed via qRT-PCR assay.

### Cell transfection

HeLa and CaSki cells were transfected with siRNA against circ_0020460 (si-circ_0020460, ACCGTCCGGGTGAAGGAAGTT) (Ribobio, Guangzhou, China), miR-485-3p mimics (Ribobio), miR-485-3p inhibitors (Ribobio), and pcDNA plasmid expressing CXCL1 (C-X-C motif chemokine ligand 1) (GenePharma, Shanghai, China), and their matched negative control (si-con, miR-con, in-miR-con, and pcDNA) alone or jointly following the user’s manual of DharmaFECT Duo (Dharmacon, Lafayette, Colorado, USA).

### Cell counting kit-8 (CCK-8) and 5-ethynyl-2ʹ-deoxyuridine (Edu) assay

In line with the instruction of CCK-8 kit (Dojindo, Kumamoto, Japan), HeLa and CaSki cells were subjected to a certain time of treatment and passaged in 96-well dishes. The cells were then exposed to CCK-8 reagent at 37 °C. Sensitive colorimetric method was used to determine the number of viable cells. For Edu assay, HeLa and CaSki cells were subjected to certain transfections and passaged in 96-well dishes, followed by culturing at 37 °C. The following procedures were conducted as instructed by the manufacturer of EdU staining kit (Ribobio). The EdU solution used in this assay was prepared using DMEM and MACOY’S 5A medium (EK-Bioscience, Shanghai, China). The cells were fixed with paraformaldehyde and incubated with click reaction solution. Analysis of cell proliferation was performed under a fluorescence microscope (Etaluma Inc., Carlsbad, CA).

### Wound healing assay

Migration capability of CaSki and HeLa cells was detected by using wound healing assay. HeLa and CaSki cells were passaged in six-well plates and continuously cultured until the cell density reached 95%. Then, scratches were made using pipette tips. After 24 h of culture with serum-free medium (GIBCO), the rate of HeLa and CaSki cells migration of was evaluated by measuring the wound distance using Image J software.

### Transwell assay

CaSki and HeLa cells from different groups were washed and resuspended in serum-free medium. The upper chambers of 24-well plates were added with the cell suspension, whereas the appropriate inducer was added to the lower chambers. The non-migrated cells were removed using cotton swabs. The migrated cells were incubated with with methanol (Solarbio) and crystal violet (Solarbio). An inverted light microscope was applied to observe the staining results. The upper chambers of transwell inserts were precoated with Matrigel (Solarbio) in advance for invasion assay.

### Flow cytometry assay

The apoptotic rate of CC cells was assessed by Annexin V-FITC/PI (Solarbio, Beijing, China). In brief, transfected HeLa and CaSki cells were injected into 6-well plates and cold PBS (Solarbio) was used to wash cells. After staining the cells with AnnexinV-FITC and PI, and cells samples were subsequently assessed using a flow cytometer. (BD Biosciences, Franklin Lake, NJ, USA).

### Western blot assay

Cell homogenates were prepared using a lysis buffer containing aprotinin, leupeptin, sodium orthovanadate, sodium pyrophosphate and EDTA (Beyotime). Samples were incubated at 4 °C and supernatant was collected by centrifugation. The Bradford assay method was used for concentration analysis of protein samples. Proteins were separated on SDS-PAGE gels and transferred onto PVDF (Millipore, Billerica, MA, USA) membranes. The following primary antibodies were used for membrane incubation, including against β-actin (1:2000, ab8227, Abcam, Cambridge, UK), CXCL1 (1:1000, ab86436, Abcam), BAX (ab216494, 1:1000), and BCL2 (ab32124, 1:1000). After that, the membranes were maintained 2 h with secondary antibody (bs-0370R-HRP; Bioss, Beijing, China). At last, membrane-bound antibodies were analyzed using eyoECL Plus (Beyotime).

### Tube formation assay

The capillary-like network formation was used to analyze angiogenic capacity. In short, the conditioned culture medium of HeLa and CaSki cells with different transfections was collected. Then, HUVECs in the conditioned culture medium were plated into 96-well plates coated with growth factor-depleted Matrigel (Corning; Bedford, MA, USA). After incubation, the angiogenic capacity were analyzed using image J software.

### Bioinformatics analysis and Dual-luciferase reporter assay

Circular RNA interactome was used for predicting miRNA targets for circ_0020460, and microT CDS was used for predicting the target gene of miR-485-3p. The luciferase plasmids were constructed by using Luciferase vector pmirGLO (Promega, Madison, WI, USA). The wild-type and mutant-type of circ_0020460 and CXCL1 3ʹUTR (circ_0020460-WT, circ_0020460-MUT, CXCL1-3ʹUTR-WT and CXCL1-3ʹUTR-MUT) plasmids were co-transfected with miR-con or miR-485-3p into CaSki and HeLa cells, respectively. The cells were cultured to allow gene expression, and the luminescence was analyzed using Dual-Lucy Assay Kit (Solarbio).

### RNA immunoprecipitation (RIP) assay

In accordance with the supplier’s protocol of Magna RIP Kit (Abcam), the assay was performed using anti-Ago2 (Abcam) and anti-IgG (Abcam). First of all, cell lysates of CaSki and HeLa cells were prepared using RIPA buffer and then exposed to magnetic beads coated with the above mentioned antibodies. The co-precipitated RNA was isolated, and the circ_0020460, miR-485-3p, and CXCL1 mRNA level were analyzed by qRT-PCR.

### Tumor xenograft assay

Five-week-old male BALB/c nude mice (Hunan Slyke Jingda Experimental Animal Co., LTD, Changsha, China) for this xenograft assay were randomly divided into 3 groups and housed in animal facilities with available food and water. The mice were subcutaneously injected with CaSki cells (5 × 10^6^) infected with lentiviral-packaged sh-circ_0020460, sh-con or CaSki cells (Empty). Tumor sizes were measured every one week, and mice were euthanatized after a 28-day experimental period. The tumor tissues of mice were collected and frozen in liquid nitrogen. The Animal Care and Use Committee of Northwest Women’s and Children’s Hospital approved the animal study. All animal procedures were carried out following the guidelines of the National Animal Care and Use of laboratory animals, the ARRIVE guidelines and the Basel Declaration.

### Immunohistochemistry assay

First, dewaxed and hydrated the paraffin sections, then heated with 3% H_2_O_2_. Sections were rinsed with distilled water and PBS, followed by antigen retrieval through microwave and serum blocking. Incubate slides with anti-Ki67 (1:1000, ab15580, Abcam), anti-BAX (1: 100, ab216494, Abcam), anti-BCL2 (1:100, ab138498, Abcam) at 4 °C overnight, then incubated with the secondary antibody (goat Anti-Rabbit IgG H&L (HRP), 1:2,000, ab6721, Abcam) at room temperature. The stains were visualized by using DAB (Vector Laboratories, Peterborough, UK). After rinsed in tap water, dehydrated, cleared, and sealed, the sections were subjected to image capture using a microscope (Olympus). ImageJ software was employed for semi-quantitative analysis of the staining results, and the positive expression rate was calculated.

### Statistical analysis

All results were expressed as mean ± standard deviation (SD) and analyzed by GraphPad Prism software. Each cell experiment was performed at least three times. Analysis of variance or Student’s *t*-test was used to analyze the relevant data. The Pearson correlation coefficient was used to analyze the linear correlation between any two of circ_0020460, miR-485-3p and CXCL1. *P* < 0.05 indicated the statistical difference.

## Results

### Circ_0020460 level was increased in CC tissues and cell lines

**Fig. 1:**
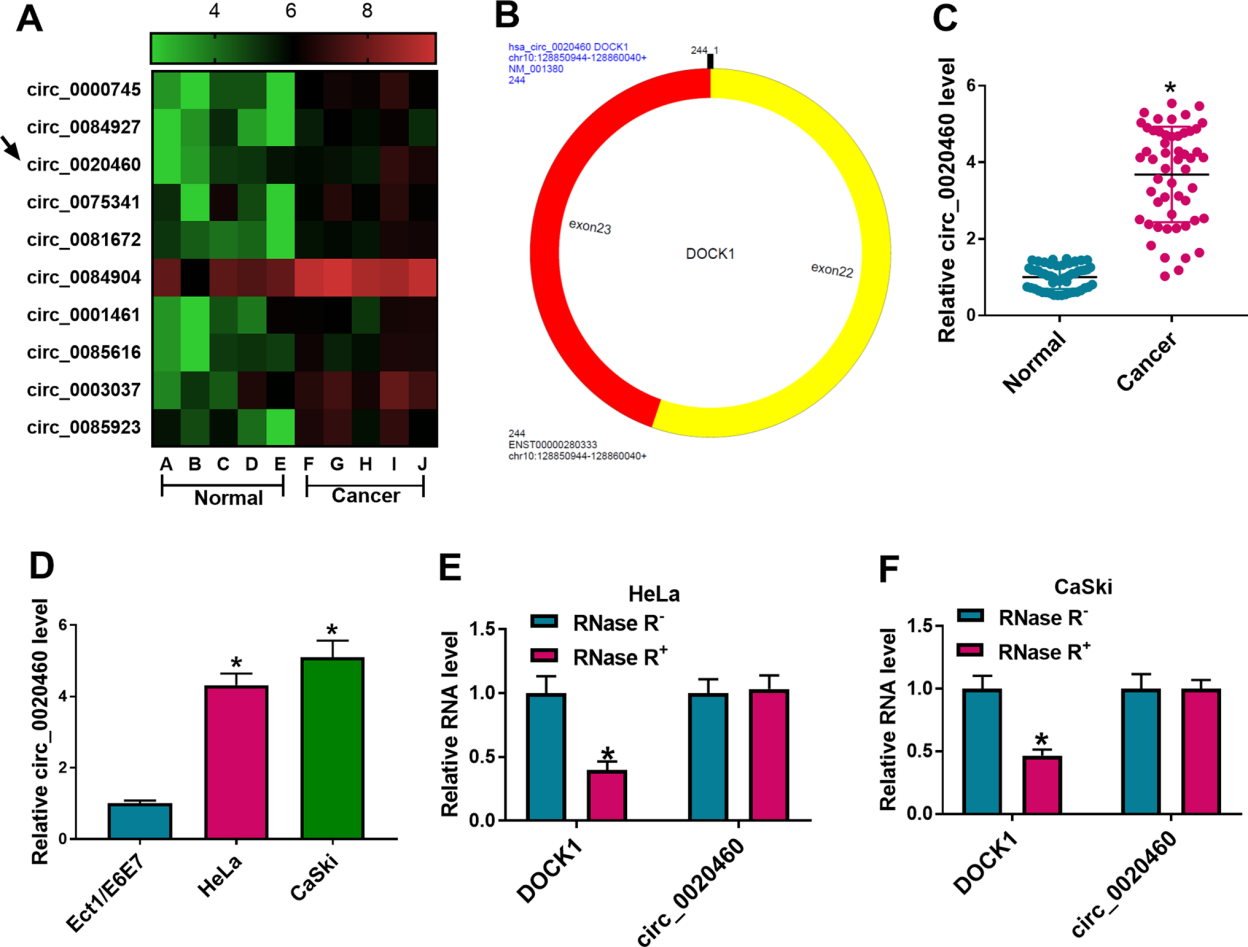
Upregulation of circ_0020460 in CC tissues and cell lines. **A** The analysis results of circ_0020460 expression in GSE102686 dataset. **B** The genic information of circ_0020460. **C** The levels of circ_0020460 in CC tissues and normal tissues were detected by qRT-PCR. **D** The levels of circ_0020460 in Ect1/E6E7, HeLa and CaSki cells were detected by qRT-PCR. **E**, **F** The levels of DOCK1 and circ_0020460 in HeLa and CaSki cells treated with or without RNase R were detected by qRT-PCR. The t-test (**C**), one-way ANOVA (**D**), and two-way ANOVA (**E**, **F**) were used. *P < 0.05

We revealed that circ_0020460 was overexpressed in CC tissues than that in normal tissues in GSE102686 dataset (Fig. 1A). Figure 1B displayed the genic information of circ_0020460. Circ_0020460 was derived from exon 22–23 of DOCK1 with the mature length of 244 bp. qRT-PCR evaluated that circ_0020460 level was dramatically increased in CC tissues (Fig. 1C). The level of circ_0020460 was also up-regulated in Hela and CaSki cells compared with that in Ect1/E6E7 cells (Fig. 1D). The stability of circ_0020460 was investigated by qRT-PCR. In Hela and CaSki cells, after RNase R treatment, the DOCK1 mRNA was prominently reduced, while circ_0020460 was resistant to RNase R (Fig. 1E–F). These results demonstrated that circ_0020460 was dysregulated in CC.

### Circ_0020460 knockdown induced cell apoptosis but blocked cell proliferation, migration, invasion, and angiogenesis in Hela and CaSki cells

**Fig. 2:**
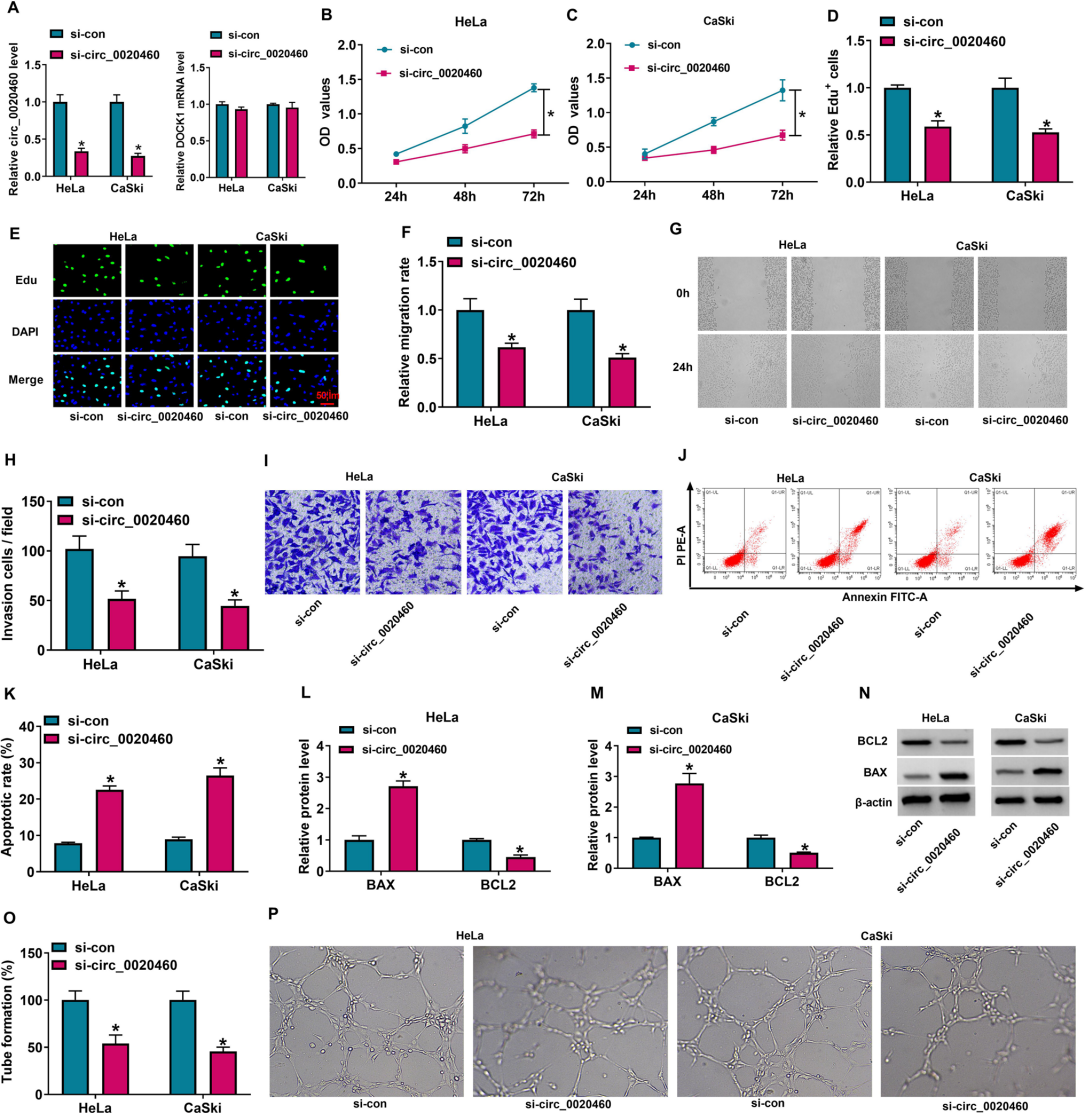
Effects of circ_0020460 on proliferation, migration, invasion, apoptosis and angiogenesis. **A** The expression of circ_0020460 and DOCK1 mRNA in HeLa and CaSki cells transfected with si-con or si-circ_0020460 was detected by qRT-PCR. **B**–**E** Cell proliferation in HeLa and CaSki cells after si-con or si-circ_0020460 transfection was assessed by CCK-8 assay (**B**, **C**) and Edu assay (**D**, **E**). **F**–**I** Cell migration and cell invasion in HeLa and CaSki cells after si-con or si-circ_0020460 transfection was assessed by wound healing assay (**F**, **G**) and transwell assay (**H**, **I**). **J**, **K** HeLa and CaSki cell apoptosis was analyzed by flow cytometry analysis. **L**–**N** The protein levels of BAX and BCL2 in transfected HeLa and CaSki cells were measured by western blot assay, respectively. **O**, **P** The angiogenic ability was detected by tube formation assay. The two-way ANOVA was used in **A**–**O**. *P < 0.05

Firstly, the efficiency of silencing circ_0020460 expression was detected in Hela and CaSki cells. The expression of circ_0020460 was significantly decreased in si-circ_0020460 group than that in si-con group, however, si-circ_0020460 does not alter DOCK1 mRNA expression (Fig. 2A). In function, CCK-8 assay showed that Hela and CaSki cells with si-circ_0020460 transfection had lower OD values (Fig. 2B, C). Edu assay presented that circ_0020460 knockdown reduced Edu-positive cells (Fig. 2D, E). These data suggested that si-circ_0020460 transfection slowed down the proliferation of Hela and CaSki cells. Furthermore, wound healing assay and transwell assay presented that si-circ_0020460 signally weakened migration rate and invasion number of Hela and CaSki cells (Fig. 2F–I). In addition, si-circ_0020460 also promoted cell apoptotic rate (Fig. 2J, K). Furthermore, through the western blot assay, we found that the BAX protein level was increased, while the BCL2 protein level was markedly decreased in Hela and CaSki cells transfected with si-circ_0020460 (Fig. 2L–N). Moreover, transfecting with si-circ_0020460 in Hela and CaSki cells inhibited angiogenesis through tube formation assay (Fig. 2O, P). Comprehensively, circ_0020460 knockdown repressed the malignant development of CC cells.

### MiR-485-3p was a target of circ_0020460

**Fig. 3:**
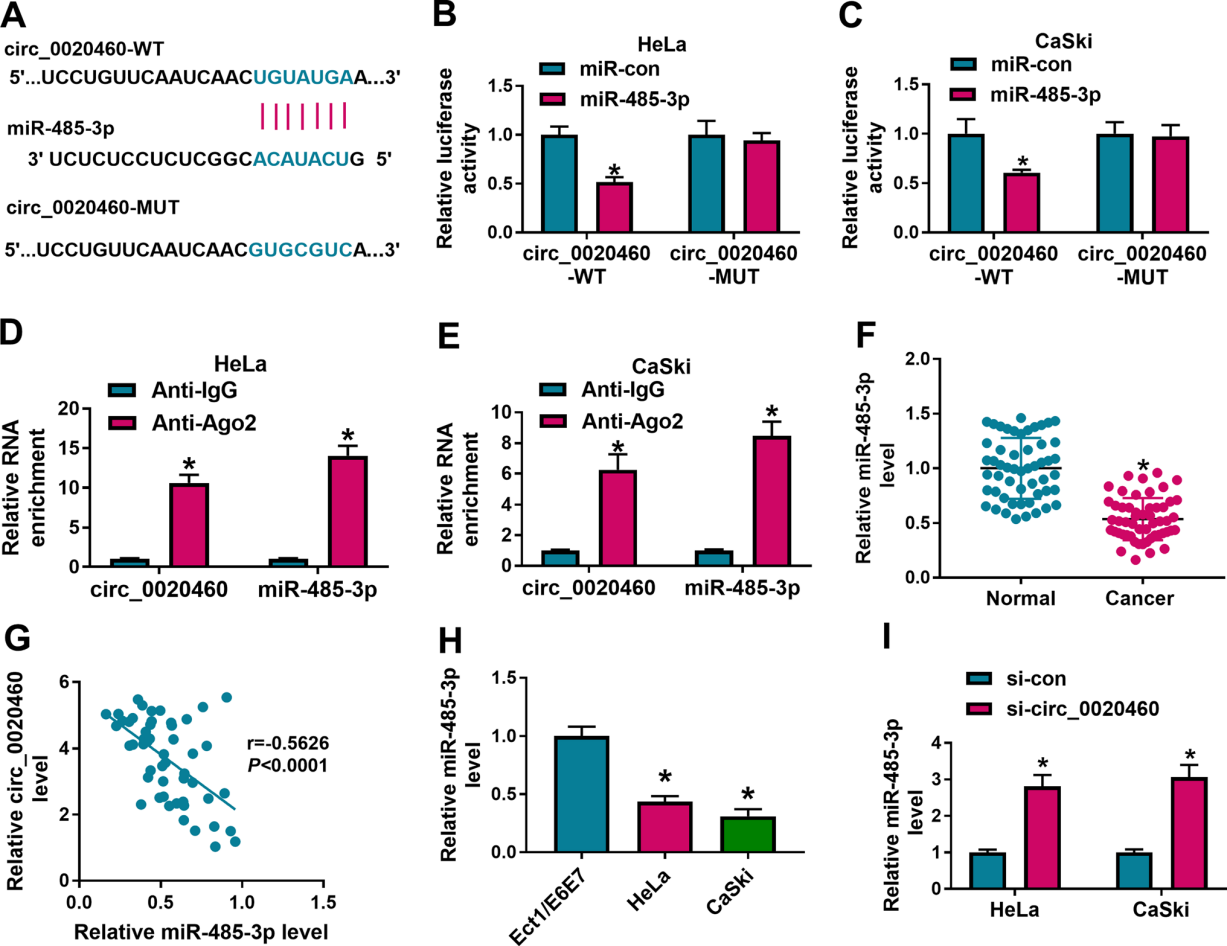
MiR-485-3p acted as the target of circ_0020460. **A** The complementary sequences between circ_0020460 and miR-485-3p were displayed. **B**–**E** The interaction between circ_0020460 and miR-485-3p was analyzed by dual-luciferase reporter assay and RIP assay. **F** The expression of miR-485-3p in CC tissues and normal tissues was detected by qRT-PCR. **G** The correlation between the levels of miR-485-3p and circ_0020460 in CC tissues was analyzed by pearson correlation coefficient analysis. **H** The level of miR-485-3p in Ect1/E6E7, HeLa and CaSki cells was detected by qRT-PCR. **I** The expression of miR-485-3p in HeLa and CaSki cells transfected with si-con or si-circ_0020460 was detected by qRT-PCR. The t-test (**F**), one-way ANOVA (**H**) and two-way ANOVA (**B**–**E**, **I**) were used. *P < 0.05

MiR-485-3p was predicted as a target of circ_0020460 by online software circular RNA interactome (https://circinteractome.nia.nih.gov/) and the binding sites between circ_0020460 and miR-485-3p were shown in Fig. 3A. In addition, dual-luciferase reporter assay showed that the cotransfection of circ_0020460-WT and miR-485-3p in Hela and CaSki cells inhibited luciferase activity (Fig. 3B, C). Furthermore, both circ_0020460 and miR-485-3p could be abundantly enriched in the Anti-Ago2 group compared with Anti-IgG group through RIP assay (Fig. 3D, E). In CC patients, miR-485-3p level was lower in cancer tissues than paired normal tissues (Fig. 3F), and circ_0020460 expression was negatively and linearly correlated with miR-485-3p in cancer tissues (r = -0.5626, *P* < 0.0001) (Fig. 3G). Moreover, miR-485-3p expression level was lower in Hela and CaSki cells than that in Ect1/E6E7 cells (Fig. 3H), and transfection with si-circ_0020460 upregulated miR-485-3p expression in Hela and CaSki cells (Fig. 3I). These data demonstrated that miR-485-3p was downregulated in CC and it was a target of circ_0020460.

### Circ_0020460 knockdown inhibit the growth of CC cells by upregulating miR-485-3p

**Fig. 4:**
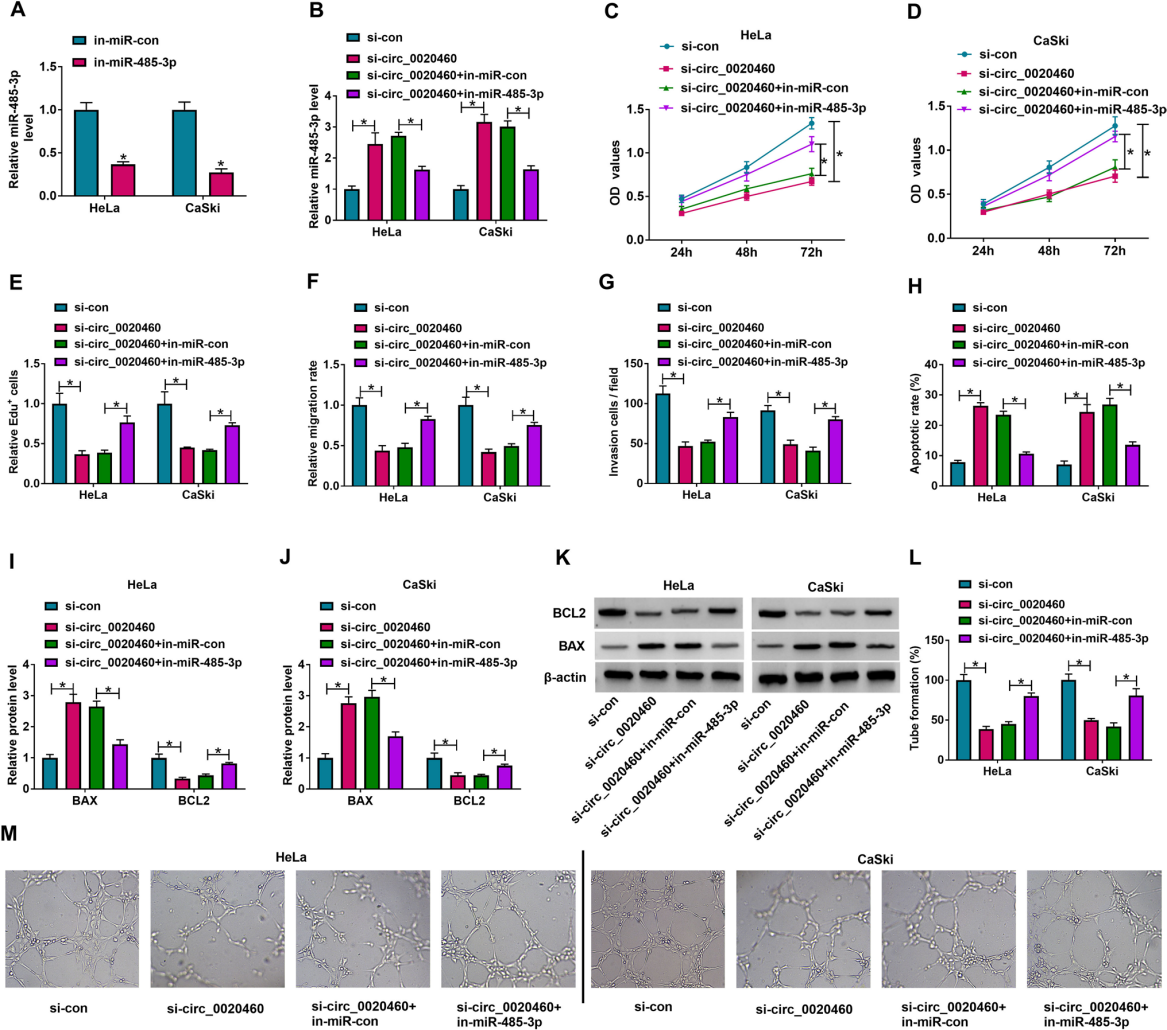
MiR-485-3p inhibitor reversed the effects of si-circ_0020460. **A** The level of miR-485-3p in HeLa and CaSki cells transfected with in-miR-con and in-miR-485-3p was detected by qRT-PCR. **B**–**L** HeLa and CaSki cells were treated with si-con, si-circ_0020460, si-circ_0020460 + in-miR-con, or si-circ_0020460 + in-miR-485-3p. **B** The level of miR-485-3p in HeLa and CaSki cells was detected by qRT-PCR. **C**–**E** Cell proliferation in HeLa and CaSki cells after transfection was assessed by CCK-8 assay (**C**, **D**) and Edu assay (**E**). **F**, **G** Cell migration and cell invasion in HeLa and CaSki cells after transfection was assessed by wound healing assay (**F**) and transwell assay (**G**). **H** HeLa and CaSki cells apoptosis was analyzed by flow cytometry analysis. **I**–**K** The protein levels of BAX and BCL2 in transfected HeLa and CaSki cells were measured by western blot assay, respectively. **L**, **M** The angiogenic ability was detected by tube formation assay. The two-way ANOVA was used in **A**–**L**. *P < 0.05

A series of rescue experiments were carried out to understand whether circ_0020460 regulate CC development by mediating miR-485-3p. The expression of miR-485-3p was notably decreased in Hela and CaSki cells transfected with miR-485-3p inhibitor (Fig. 4A). When Hela and CaSki cells transfected with si-circ_0020460, the miR-485-3p expression was elevated, while the expression of miR-485-3p was partly recovered in si-circ_0020460 + miR-485-3p inhibitor group (Fig. 4B). In Hela and CaSki cells, cotransfection of si-circ_0020460 and miR-485-3p inhibitor largely reversed the inhibitory effect of si-circ_0020460 on cell proliferation. (Fig. 4C–E). Cell migration and invasion was inhibited in Hela and CaSki cells transfected with si-circ_0020460, but was enhanced in cells cotransfected with si-circ_0020460 and miR-485-3p inhibitor relative to si-circ_0020460 + in-miR-con (Fig. 4F, G). A promoting trend of cell apoptosis was measured in Hela and CaSki cells transfected with si-circ_0020460 than si-con, and cotransfection of si-circ_0020460 and in-miR-485-3p in Hela and CaSki cells reversed the promotion of cell apoptosis compared to si-circ_0020460 + in-miR-con (Fig. 4H). In addition, the inhibitor of miR-485-3p reversed the effects of si-circ_0020460 on BCL2 and BAX levels in Hela and CaSki cells (Fig. 4I–K). Moreover, tube formation assay showed that transfected with si-circ_0020460 in Hela and CaSki cells inhibited angiogenesis, while si-circ_0020460 + in-miR-485-3p reversed this effect (Fig. 4L, M). In short, miR-485-3p inhibitor reversed the effects of si-circ_0020460 on CC cell progression, indicating that si-circ_0020460 blocked CC malignant development by promoting miR-485-3p expression.

### CXCL1 was a target gene of miR-485-3p

**Fig. 5:**
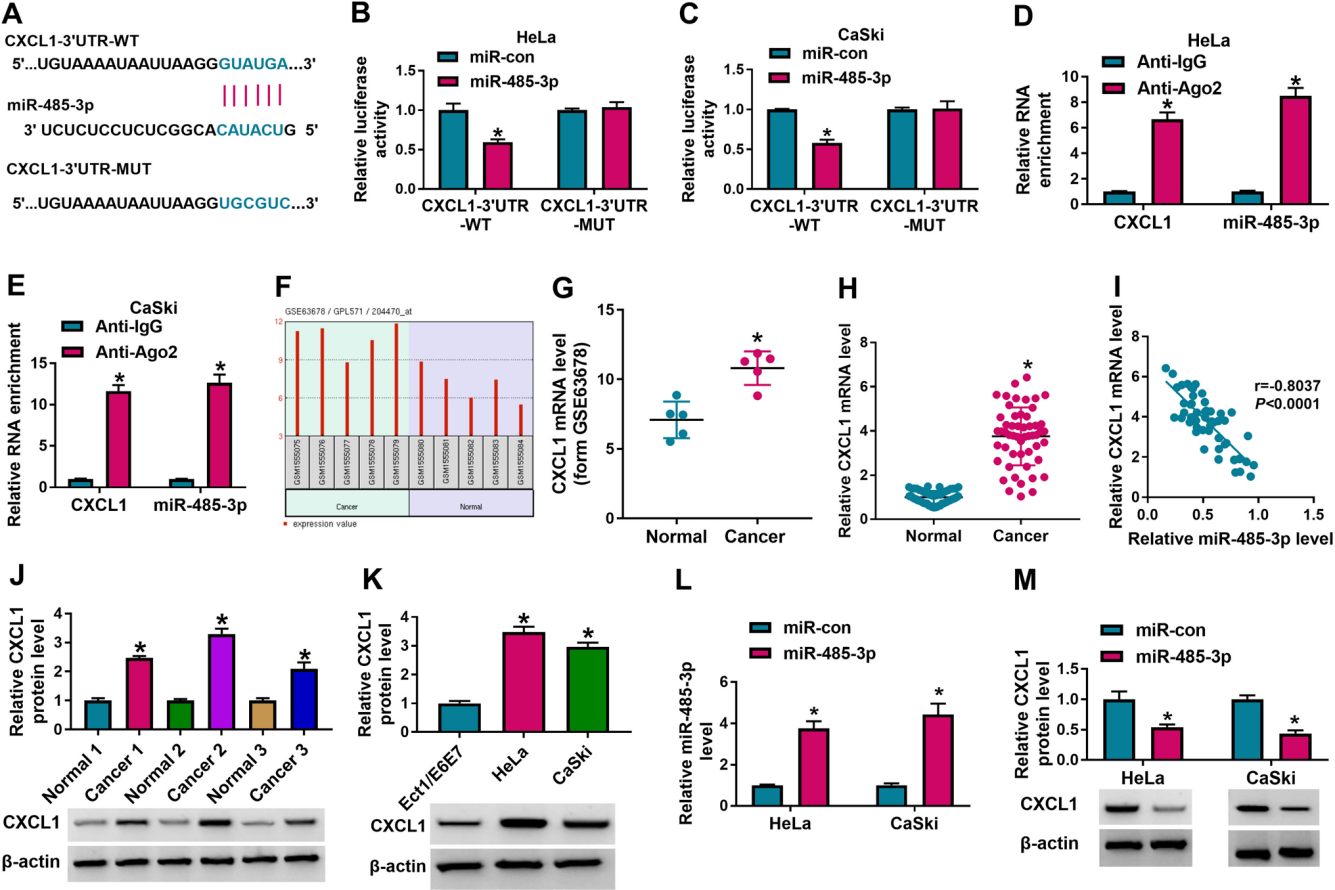
CXCL1 was the target gene of miR-485-3p. **A** The binding sites between CXCL1 and miR-485-3p. **B**–**E** The interaction between miR-485-3p and CXCL1 was verified by dual-luciferase reporter assay and RIP assay. **F**, **G** The analysis results of CXCL1 expression in GSE63678 dataset. **H** The mRNA level of CXCL1 in CC tissues and normal tissues was determined by qRT-PCR. **I** The linear correlation between CXCL1 mRNA and miR-485-3p was estimated. **J**, **K** The protein level of CXCL1 in CC tissues and cell lines was measured by western blot assay. **L**, **M** The level ofmiR-485-3p and CXCL1 protein in HeLa and CaSki cells transfected with miR-con or miR-485-3p was measured via qRT-PCR and western blot assay. The t-test (**G**, **H**), one-way ANOVA (**J**, **K**) and two-way ANOVA (**B**–**E**, **L**, **M**) were used. *P < 0.05

Moreover, microT CDS (http://microrna.gr/microT-CDS/?r=microT_CDS/index) online software forecasted that miR-485-3p might bind to CXCL1 3ʹUTR, and Fig. 5A showed the putative binding site. Then, dual-luciferase reporter assay and RIP assay were performed to verify the relationship between CXCL1 and miR-485-3p. The cotransfection with CXCL1-3’UTR-WT and miR-485-3p in Hela and CaSki cells inhibited the luciferase activity relative to the cotransfection with miR-con and CXCL1-3’UTR-WT, while in circ_0020460-MUT-transfected cells had no this effect (Fig. 5B, C). RIP revealed that CXCL1 mRNA and miR-485-3p were significantly enriched by anti-Ago2 rather than anti-IgG (Fig. 5D, E). Besides, through analysis of the GEO dataset GSE63678, it was found that CXCL1 mRNA level was upregulated in CC tissues compared with normal tissues (Fig. 5F, G). In addition, CXCL1 mRNA level was also upregulated in CC tissues than that in normal tissues in the sample we collected (Fig. 5H) and expression of CXCL1 mRNA and miR-485-3p was negatively and linearly correlated (Fig. 5I). Also, the expression of CXCL1 protein was upregulated in CC tissues (Fig. 5J) and Hela and CaSki cells (Fig. 5K). The overexpression efficiency of miR-485-3p was determined by qRT-PCR, and the miR-485-3p level markedly increased (Fig. 5L) while the CXCL1 protein expression was markedly decreased (Fig. 5M). These results indicated that miR-485-3p negatively regulated CXCL1.

### MiR-485-3p overexpression boycotted cancer properties by downregulating CXCL1 of CC cells

**Fig. 6:**
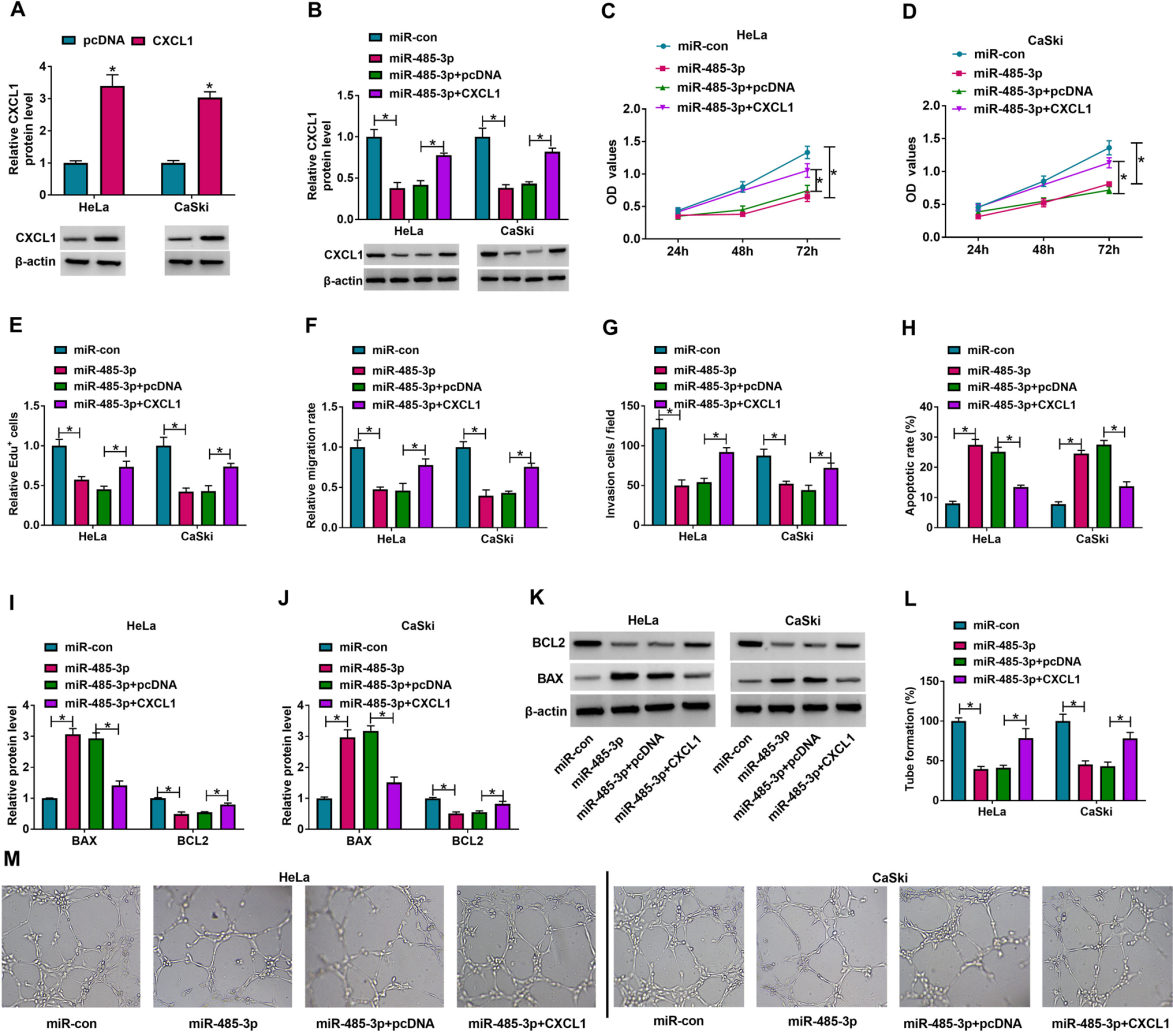
MiR-485-3p regulated CC cells progression by regulating CXCL11. **A** The protein level of CXCL1 in HeLa and CaSki cells transfected with pcDNA or CXCL1 was measured by western blot assay. **B**–**L** HeLa and CaSki cells were treated with miR-con, miR-485-3p, miR-485-3p + pcDNA or miR-485-3p + CXCL1. **B** The level of CXCL1 protein in HeLa and CaSki cells was detected by western blot assay. **C**–**E** Cell proliferation in HeLa and CaSki cells after transfection was assessed by CCK-8 assay (**C**, **D**) and Edu assay (**E**). **F**, **G** Cell migration and cell invasion in HeLa and CaSki cells after transfection was assessed by wound healing assay (**F**) and transwell assay (**G**). **H** HeLa and CaSki cell apoptosis was analyzed by flow cytometry analysis. **I**–**K** The protein levels of BAX and BCL2 in transfected HeLa and CaSki cells were measured by western blot assay, respectively. **L**, **M** The angiogenic ability was detected by tube formation assay. The two-way ANOVA was used in **A**–**L**. *P < 0.05

Firstly, the expression level of CXCL1 protein was determined by western blot, and the CXCL1 protein level markedly increased (Fig. 6A). Likewise, as Fig. 6B displayed, miR-485-3p mimic apparently downregulated the protein level of CXCL1, while CXCL1 and miR-485-3p cotransfection restored the effects. CCK-8 and Edu analysis showed that miR-485-3p up-regulation reduced cell proliferation, while CXCL1 overexpression reversed the effects (Fig. 6C–E).

Wound healing and transwell assay revealed that miR-485-3p reintroduction significantly reduced the ability of cell migration and invasion, which this effect was abrogated by overexpressing CXCL1 (Fig. 6F, G). Flow cytometry assay demonstrated miR-485-3p overexpression strikingly induced the apoptotic rate, which was reversed by cotransfecting with CXCL1 and miR-485-3p in Hela and CaSki cells (Fig. 6H). Moreover, miR-485-3p mimic remarkably increased BAX expression, and decreased BCL2 protein level, whereas these effects were attenuated by overexpressing CXCL1 (Fig. 6I–K). Finally, tube formation assay indicated that transfected with miR-485-3p in Hela and CaSki cells inhibited angiogenesis, while cotransfection with CXCL1 and miR-485-3p reversed this effect (Fig. 6L, M). All these data reflected that overexpression of CXCL1 abolished the effects of miR-485-3p overexpression on progression of Hela and CaSki cells.

### Circ_0020460 indirectly regulates CXCL1 via miR-485-3p in CC cells

**Fig. 7:**
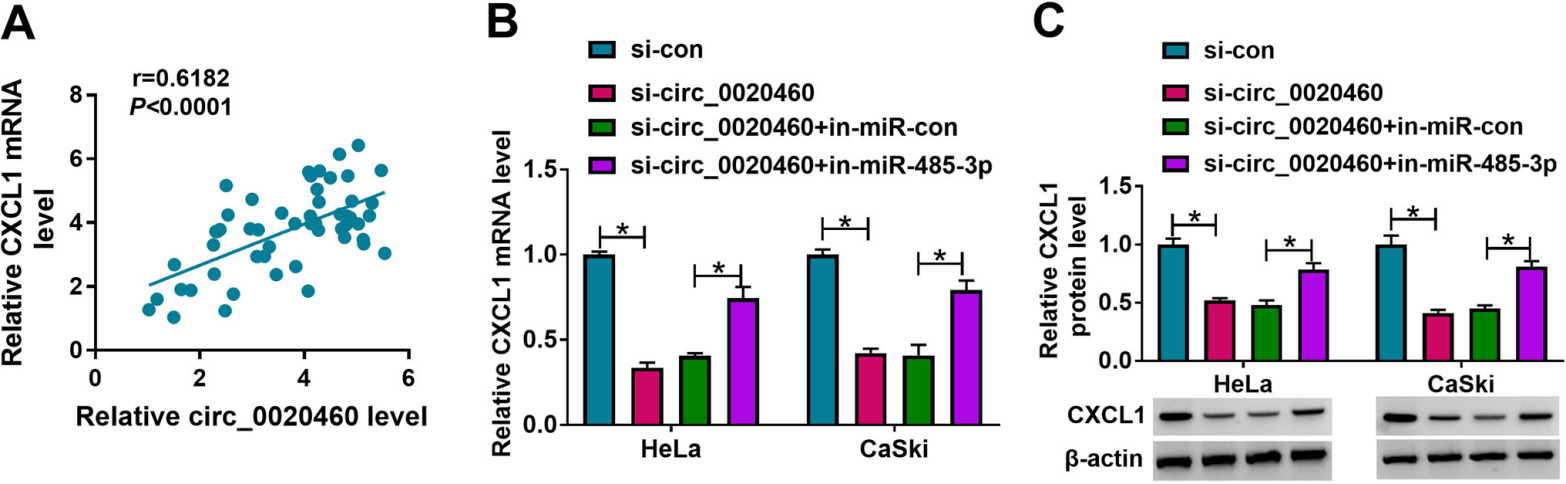
Circ_0020460 regulated CXCL1 expression by targeting miR-485-3p. **A** The correlation between the levels of circ_0020460 and CXCL1 in CC tissues was analyzed. **B**, **C** The protein level of CXCL1 in HeLa and CaSki cells transfected with si-con, si-circ_0020460, si-circ_0020460 + in-miR-con or si-circ_0020460 + in-miR-485-3p was measured by western blot assay. The two-way ANOVA was used in **B**, **C**.*P < 0.05

Considering that circ_0020460 could sponge with miR-485-3p and CXCL1 was a target gene of CXCL1, we assumed that circ_0020460 might regulate CXCL1 expression via miR-485-3p. As exhibited in Fig. 7A, the positive correlation between circ_0020460 and CXCL1 mRNA expression (R = 0.6182, *P* < 0.0001) in the CC tissues. Additionally, western blot analysis showed miR-485-3p inhibitor overturned si-circ_0020460-mediated downregulation of CXCL1 protein expression in Hela and CaSki cells (Fig. 7B, C). In all, we confirmed that circ_0020460 could indirectly regulate CXCL1 by targeting miR-485-3p in Hela and CaSki cells.

### Knockdown of circ_0020460 inhibited tumor growth in vivo

**Fig. 8:**
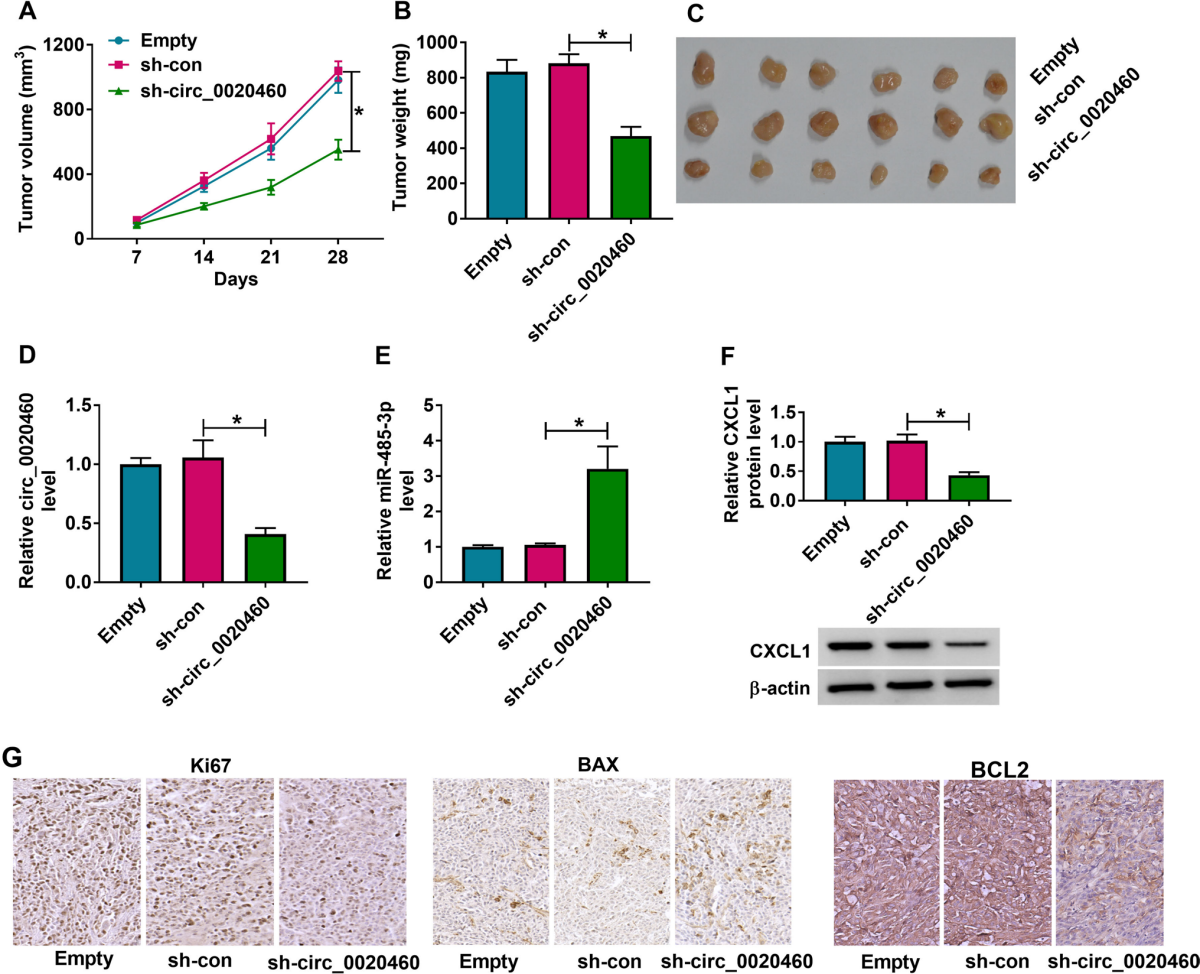
Knockdown of circ_0020460 inhibited the growth of CC cells in vivo. **A**–**C** Tumor volume and weight after circ_0020460 knockdown in vivo were measured. **D**–**F** Levels of circ_0020460, miR-485-3p and CXCL1 protein were determined. **G** IHC analysis for Ki67, BAX and BCL2 in mice tumors were conducted. The two-way ANOVA (**A**) and one-way ANOVA (**B**–**F**) and were used. *P < 0.05

Furthermore, to investigate the carcinogenic activity of circ_0020460 in vivo, CaSki cells were stably transfected with sh-circ_0020460, sh-con or without transfection to conduct xenograft model in vivo, and the data showed tumor volume and weight were greatly suppressed in sh-circ_0020460 group compared with sh-con group (Fig. 8A–C). Furthermore, qRT-PCR analysis showed circ_0020460 expression was lower, and miR-485-3p expression was higher in tumors from sh-circ_0020460 group than that in sh-con groups (Fig. 8D, E). In addition, western blot analysis showed CXCL1 protein expression was lower in tumors from sh-circ_0020460 group than that in sh-con groups (Fig. 8F). IHC assay also demonstrated decreased Ki67 and BCL2 positive signals and increased BAX positive signal in the xenogaft tumors of sh-circ_0020460 group group when compared with the sh-con group. (Fig. 8G). Therefore, we validated that knockdown of circ_0020460 impeded tumor growth in vivo.

## Discussion

Many studies have found that circRNAs play a significant functional role in CC [20]. Song et al*.* found that hsa_circRNA_101996 could promote the invasion and proliferation of CC cells [21]. CircEIF4G2 [22], circRNA8924 [23], and circSLC26A4 [24, 25] have also been confirmed to play a carcinogenic role in CC. For investigating the function of circRNA in CC, we selected circ_0020460 by analyzing the GEO public database, which was highly expressed in cervical cancer tissues.

Hsa_circ_0020460 is derived from protein dedicator of cytokinesis 1 (DOCK1) with the location at chr10:128850944–128860040. Liu et al*.* conducted the in-depth research on circDOCK1 in bladder cancer, and their research results showed that circDOCK1 promoted the growth of cancer cells in vivo and in vitro by regulating miR-132-3p/Sox5 signaling axis [17]. In addition, the study results of Zhang et al*.* confirmed that circDOCK1 promoted the cell growth by inhibiting miR-132-3p expression in colorectal cancer [18]. In summary, circDOCK1 played a carcinogenic role in at least three kinds of malignant tumors including cervical cancer, bladder cancer, and colorectal cancer, suggesting that the important involvement of circDOCK1 in the occurrence and development of malignant tumors. However, the mechanism of circDOCK1 in CC needs to be studied deeply. Here, the biological function and molecular mechanism of circDOCK1 in promoting the CC progression was explored. This may offer new ideas for the follow-up study of the pathogenesis of CC, and may also provide a new therapeutic target for clinical treatment.

The results showed that circ_0020460 could promote the proliferation and metastasis of cervical cancer cells, inhibit apoptosis, and induce angiogenesis. MiRNA was also a noncoding RNA and circRNAs could act as molecular sponges of miRNAs. In addition, miRNAs could alter the target gene expression to regulate the development of human diseases. For instance, overexpression of circAMOTL1 up-regulated AMOTL1 expression by combining miR-485-5p to promote the growth of CC cell lines [26]. It was reported that miR-485-3p, sponged by circHIPK3, was downregulated in CC and participated in CC progression through regulating FGF2 [27]. Here, the online prediction website Circular RNA Interactome combined with dual luciferase reporter and RIP assay verified that miR-485-3p was a target of circ_0020460, and miR-485-3p was lowly expressed in both CC tissues and cell lines. In addition, the negatively correlation between circ_0020460 and miR-485-3p in the tissues of CC patients was proved. And, the inhibitor of miR-485-3p could reverse the inhibitory effect of si-circ_0020460 on growth of CC cells.

Transcription factor CXCL1 expression was frequently upregulated in various cancers [28, 29]. In one study, CXCL1 was identified as a target gene for miRNA-27b-5p, and CXCL1 impaired the inhibition effect of miR-27b-5p on ovarian carcinoma cells growth [30]. Upregulation of CXCL1 in cervical cancer cells is responsible for promoting angiogenesis and tumor growth [31]. Here, we found that there were binding sites between CXCL1 and miR-485-3p, and the mRNA and protein levels of CXCL1 were enhanced in CC tissues. We hypothesized that there was a notable correlation between CXCL1 and miR-485-3p and carried out a series of functional experiments for verification. Our data presented that the reintroduction of CXCL1 in CC cells transfected with miR-485-3p abated the inhibition of miR-485-3p on cell proliferation, invasion, migration and angiogenesis, as well as the enhancement of miR-485-3p on apoptosis. Additionally, si-circ_0020460 inhibited CXCL1 expression in CC cells through regulating miR-485-3p expression.

In conclusion, circ_0020460 promoted proliferation, metastasis and angiogenesis and inhibited apoptosis in CC cells by modulating miR-485-3p/CXCL1 axis, suggesting that circ_0020460 might have a clinical significance in preventing the malignant behaviors of CC cells.

### Supplementary Information

**Additional file1:** Uncropped images for western blots.

## Data Availability

The datasets used and analyzed during the current study are available from the corresponding author upon reasonable request.

## References

[1] Buskwofie A, David-West G, Clare CA (2020). A review of cervical cancer: incidence and disparities. J Natl Med Assoc.

[2] Fang J, Zhang H, Jin S (2014). Epigenetics and cervical cancer: from pathogenesis to therapy. Tumour Biol.

[3] Wuerthner BA, Avila-Wallace M (2016). Cervical cancer: screening, management, and prevention. Nurse Pract.

[4] Wardak S (2016). Human Papillomavirus (HPV) and cervical cancer. Med Dosw Mikrobiol.

[5] Rizzo AE, Feldman S (2018). Update on primary HPV screening for cervical cancer prevention. Curr Probl Cancer.

[6] Hu Z, Ma D (2018). The precision prevention and therapy of HPV-related cervical cancer: new concepts and clinical implications. Cancer Med.

[7] Birney E, Stamatoyannopoulos JA, Dutta A, Guigo R, Gingeras TR, Consortium EP (2007). Identification and analysis of functional elements in 1% of the human genome by the ENCODE pilot project. Nature.

[8] Patop IL, Kadener S (2018). circRNAs in cancer. Curr Opin Genet Dev.

[9] Zhang HD, Jiang LH, Sun DW, Hou JC, Ji ZL (2018). CircRNA: a novel type of biomarker for cancer. Breast Cancer.

[10] Sanger HL, Klotz G, Riesner D, Gross HJ, Kleinschmidt AK (1976). Viroids are single-stranded covalently closed circular RNA molecules existing as highly base-paired rod-like structures. Proc Natl Acad Sci U S A.

[11] Kolakofsky D (1976). Isolation and characterization of Sendai virus DI-RNAs. Cell.

[12] Hsu MT, Coca-Prados M (1979). Electron microscopic evidence for the circular form of RNA in the cytoplasm of eukaryotic cells. Nature.

[13] Nigro JM, Cho KR, Fearon ER, Kern SE, Ruppert JM, Oliner JD (1991). Scrambled exons. Cell.

[14] Memczak S, Jens M, Elefsinioti A, Torti F, Krueger J, Rybak A (2013). Circular RNAs are a large class of animal RNAs with regulatory potency. Nature.

[15] Li T, Zuo X, Meng X (2021). Circ_002059 suppresses cell proliferation and migration of gastric cancer via miR-182/MTSS1 axis. Acta Biochim Biophys Sin (Shanghai).

[16] Xuan L, Qu L, Zhou H, Wang P, Yu H, Wu T (2016). Circular RNA: a novel biomarker for progressive laryngeal cancer. Am J Transl Res.

[17] Liu P, Li X, Guo X, Chen J, Li C, Chen M (2019). Circular RNA DOCK1 promotes bladder carcinoma progression via modulating circDOCK1/hsa-miR-132-3p/Sox5 signalling pathway. Cell Prolif.

[18] Zhang W, Wang Z, Cai G, Huang P (2021). Circ_DOCK1 regulates USP11 through miR-132-3p to control colorectal cancer progression. World J Surg Oncol.

[19] Wu XB, Wu YT, Guo XX, Xiang C, Chen PS, Qin W (2022). Circular RNA hsa_circ_0007990 as a blood biomarker for unruptured intracranial aneurysm with aneurysm wall enhancement. Front Immunol.

[20] Song T, Xu A, Zhang Z, Gao F, Zhao L, Chen X (2019). CircRNA hsa_circRNA_101996 increases cervical cancer proliferation and invasion through activating TPX2 expression by restraining miR-8075. J Cell Physiol.

[21] Song TF, Xu AL, Chen XH, Gao JY, Gao F, Kong XC (2021). Circular RNA circRNA_101996 promoted cervical cancer development by regulating miR-1236-3p/TRIM37 axis. Kaohsiung J Med Sci.

[22] Mao Y, Zhang L, Li Y (2019). circEIF4G2 modulates the malignant features of cervical cancer via the miR218/HOXA1 pathway. Mol Med Rep.

[23] Liu J, Wang D, Long Z, Liu J, Li W (2018). CircRNA8924 promotes cervical cancer cell proliferation, migration and invasion by competitively binding to MiR-518d-5p /519-5p family and modulating the expression of CBX8. Cell Physiol Biochem.

[24] Chen Q, Li H, Liu J (2021). Circular RNA SLC26A4 regulates the maturation of microRNA-15a in non-small cell lung cancer cells. Oncol Lett.

[25] Ji F, Du R, Chen T, Zhang M, Zhu Y, Luo X (2020). Circular RNA circSLC26A4 accelerates cervical cancer progression via miR-1287-5p/HOXA7 Axis. Mol Ther Nucleic Acids.

[26] Ou R, Lv J, Zhang Q, Lin F, Zhu L, Huang F (2020). circAMOTL1 motivates AMOTL1 expression to facilitate cervical cancer growth. Mol Ther Nucleic Acids.

[27] Wu S, Liu S, Song H, Xia J (2021). Circular RNA HIPK3 plays a carcinogenic role in cervical cancer progression via regulating miR-485-3p/FGF2 axis. J Investig Med.

[28] Pecot CV, Rupaimoole R, Yang D, Akbani R, Ivan C, Lu C (2013). Tumour angiogenesis regulation by the miR-200 family. Nat Commun.

[29] Shrestha S, Yang CD, Hong HC, Chou CH, Tai CS, Chiew MY (2017). Integrated MicroRNA-mRNA analysis reveals miR-204 inhibits cell proliferation in gastric cancer by targeting CKS1B, CXCL1 and GPRC5A. Int J Mol Sci.

[30] Liu CH, Jing XN, Liu XL, Qin SY, Liu MW, Hou CH (2020). Tumor-suppressor miRNA-27b-5p regulates the growth and metastatic behaviors of ovarian carcinoma cells by targeting CXCL1. J Ovarian Res.

[31] Zhang W, Wu Q, Wang C, Yang L, Liu P, Ma C (2018). AKIP1 promotes angiogenesis and tumor growth by upregulating CXC-chemokines in cervical cancer cells. Mol Cell Biochem.

