# Supplementary material for: Circ_0020460 drives tumorigenesis in cervical cancer through miR-485-3p sponging
Source: Discov Oncol. 2024 Mar 27;15:88. doi: 10.1007/s12672-024-00933-1 (PMC10973326; doi:10.1007/s12672-024-00933-1)

2N-BCL2
HeLa

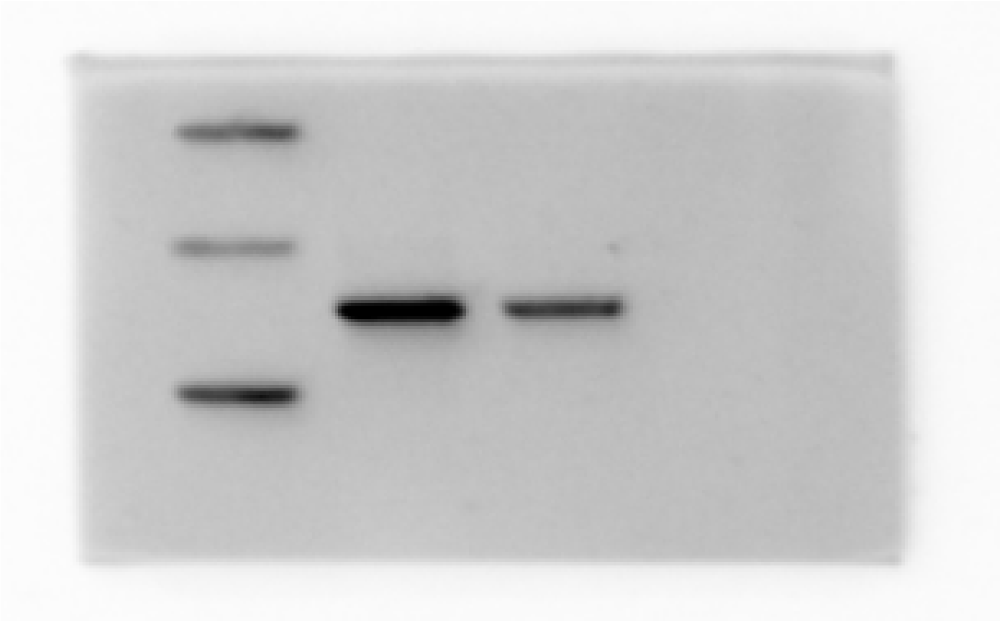

2N-BAX
HeLa

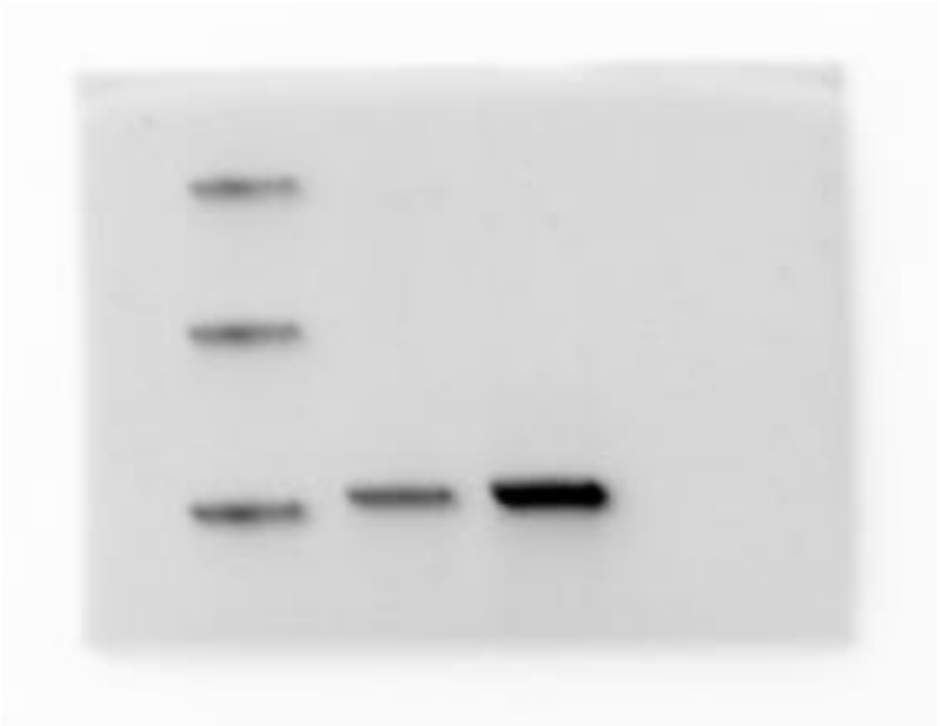

2N-β-actin
HeLa

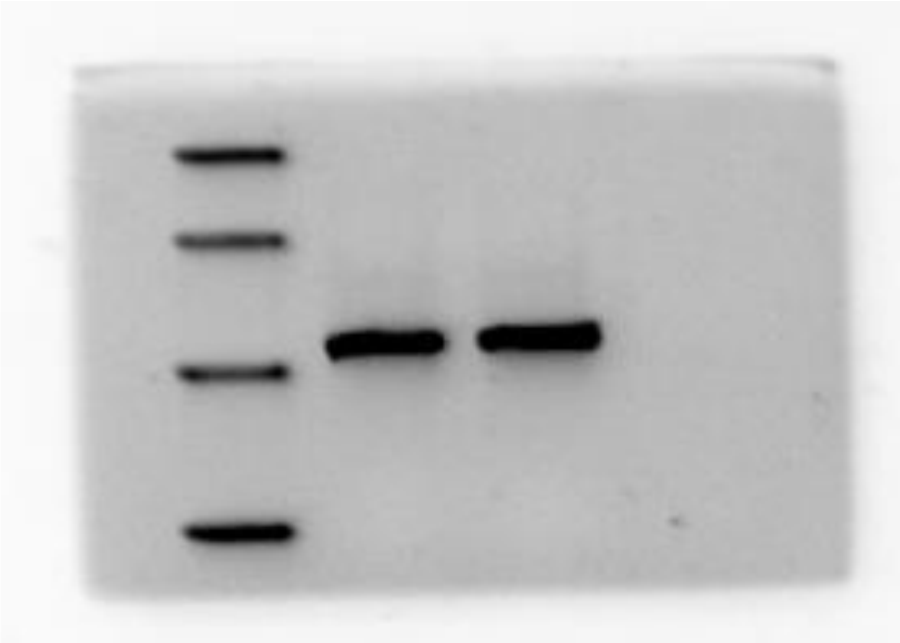

2N-BCL2
CaSki

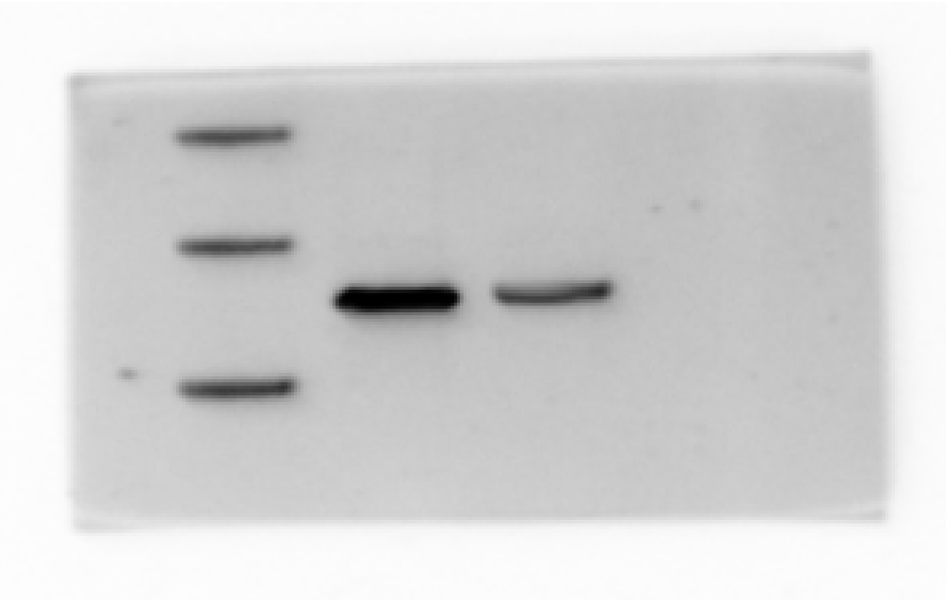

2N-BAX
CaSki

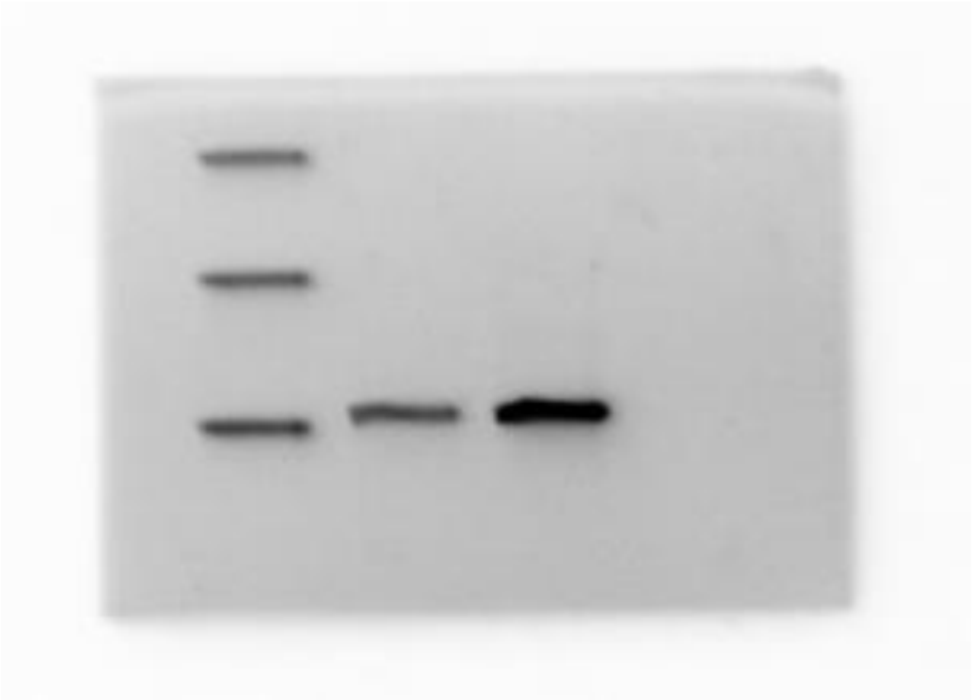

2N-β-actin
CaSki

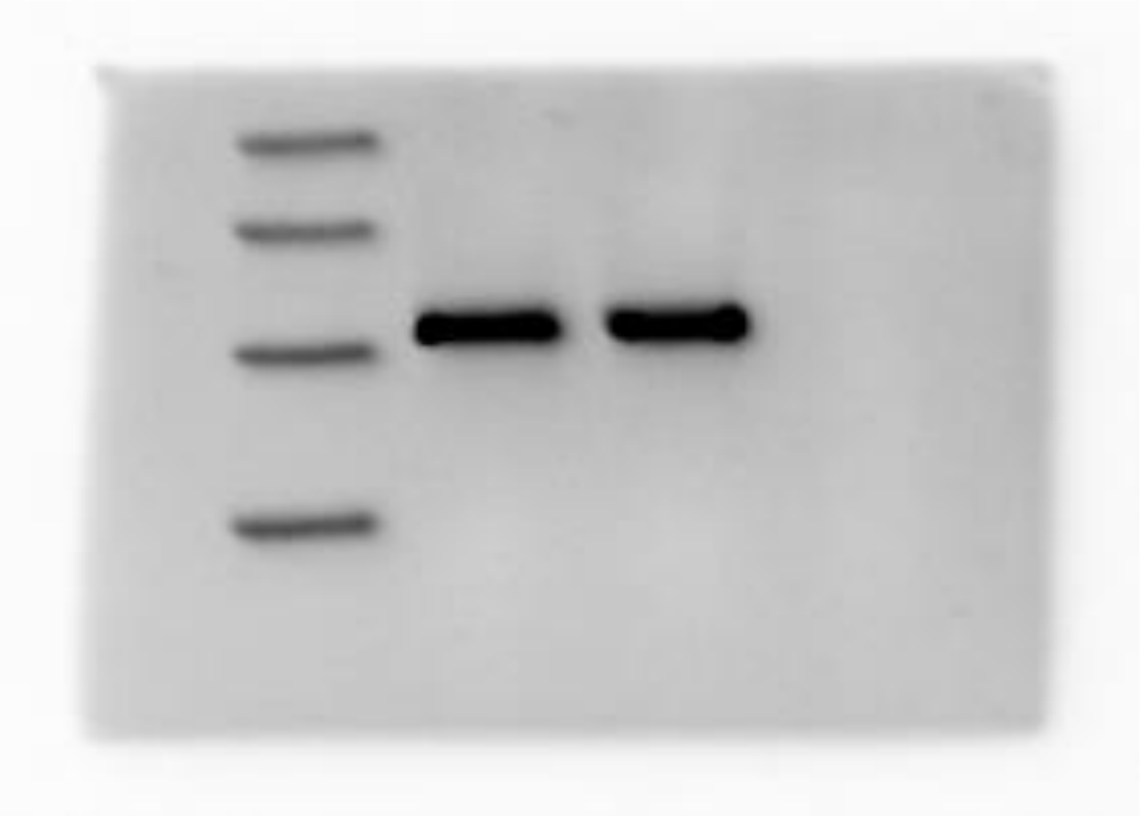

4K-BCL2
HeLa

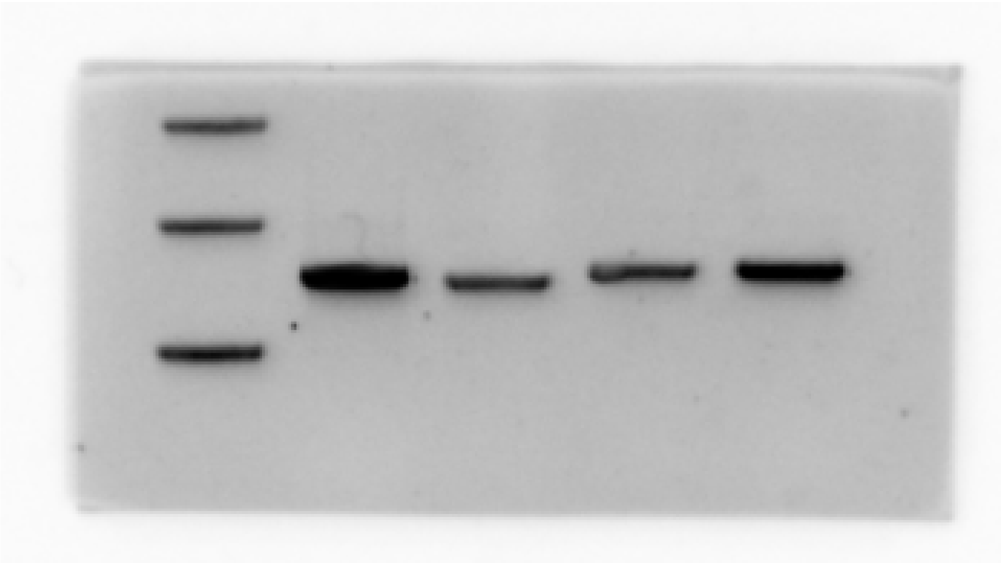

4K-BAX
HeLa

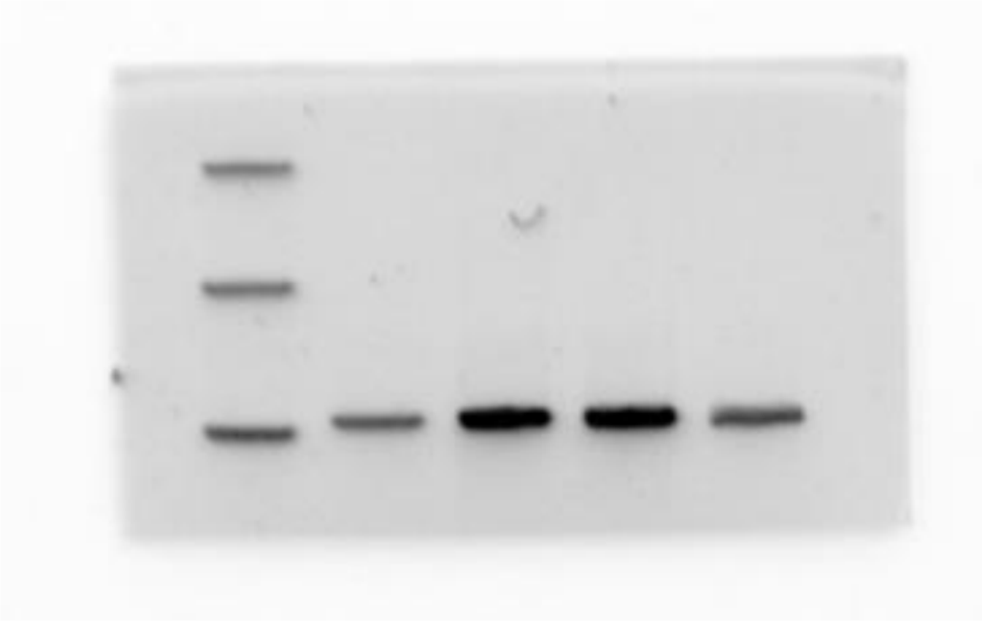

4K-β-actin
HeLa

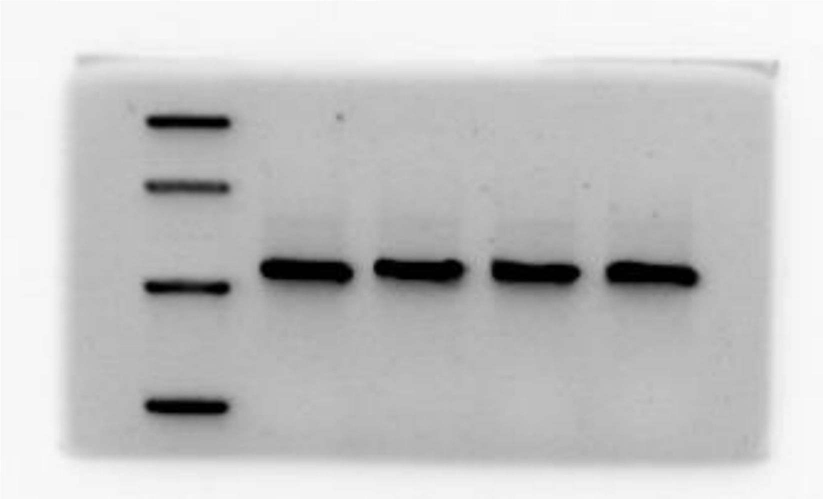

4K-BCL2
CaSki

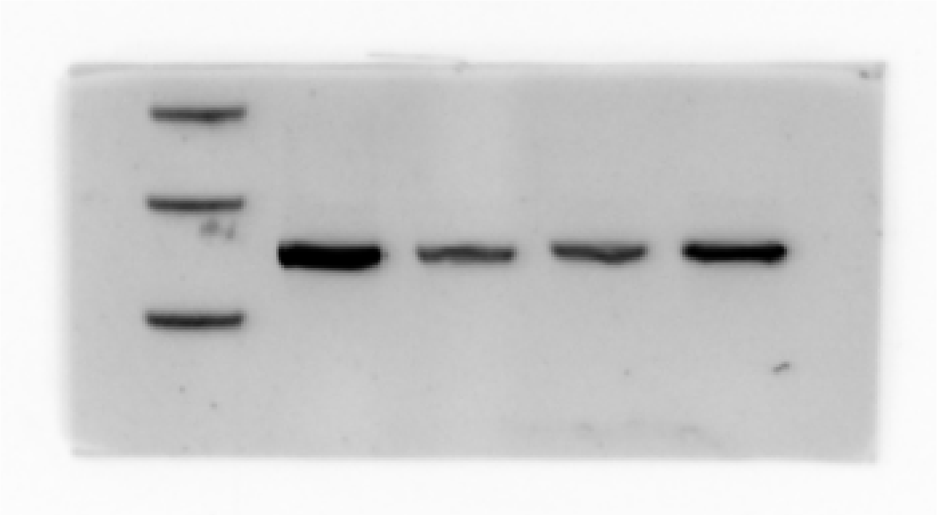

4K-BAX
CaSki

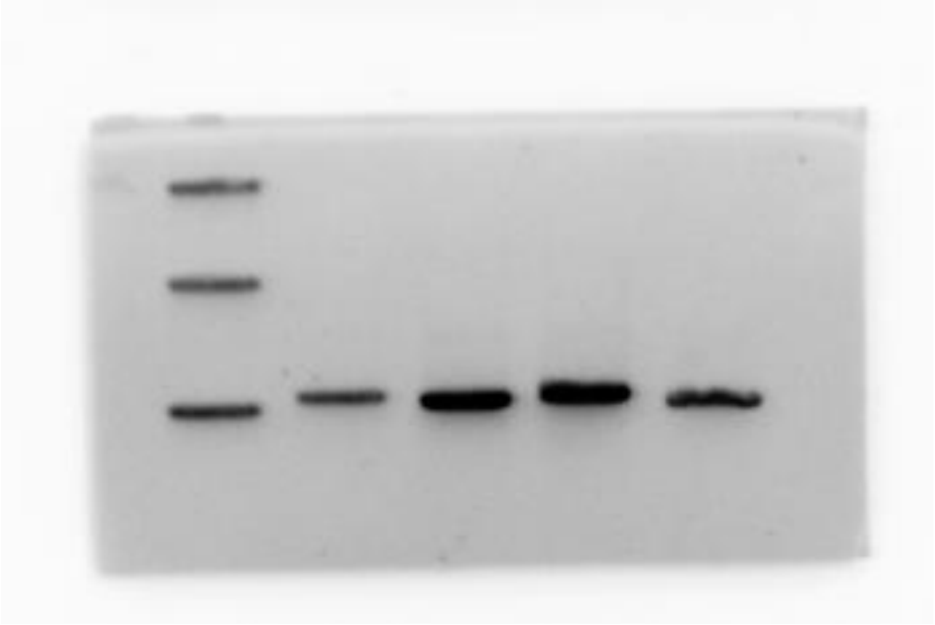

4K-β-actin
CaSki

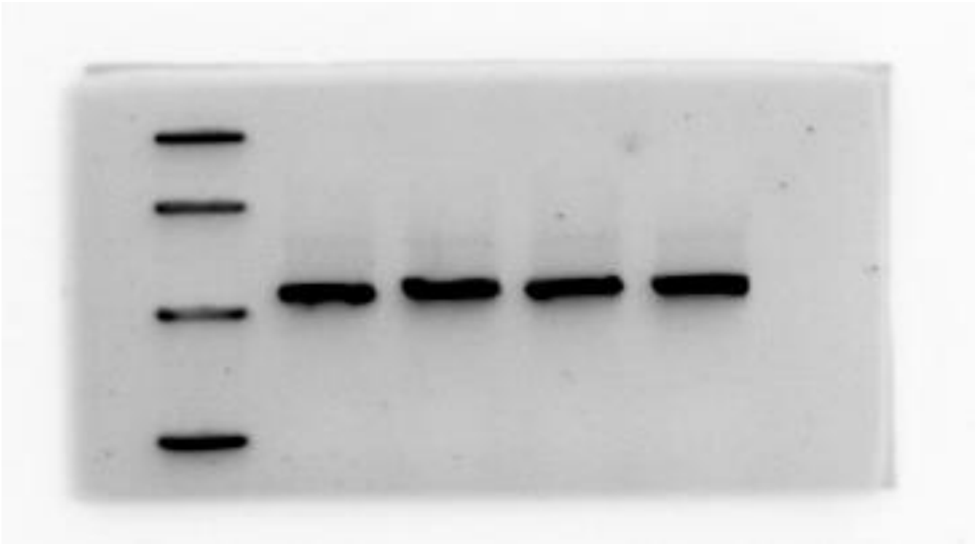

5J-CXCL1

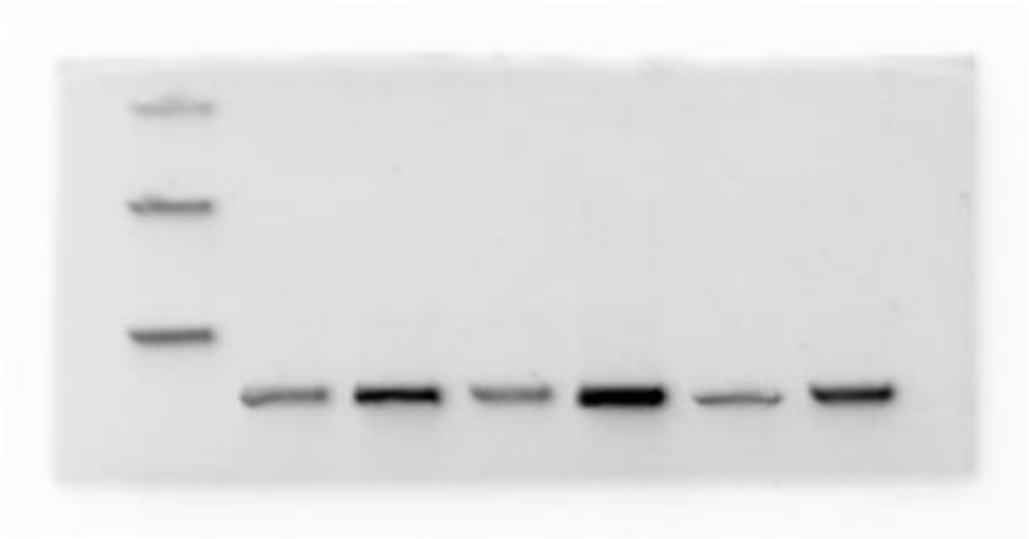

5J-β-actin

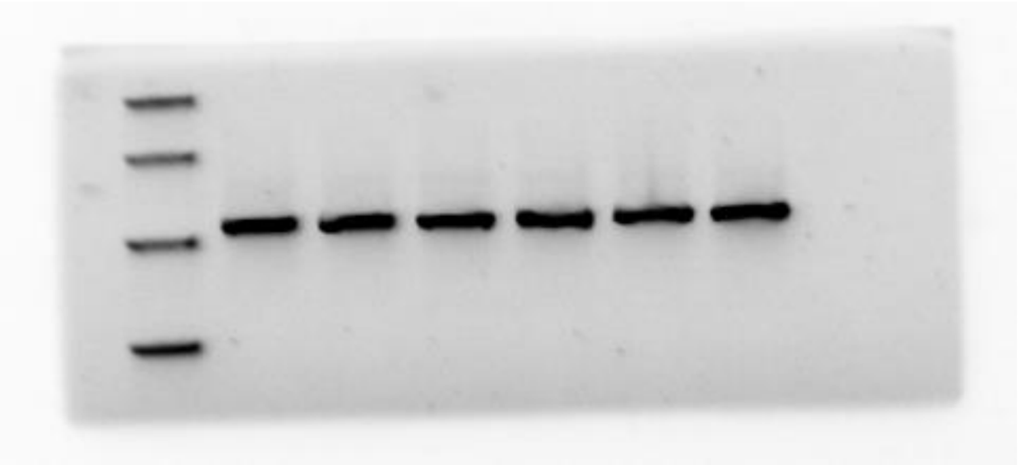

5K-CXCL1

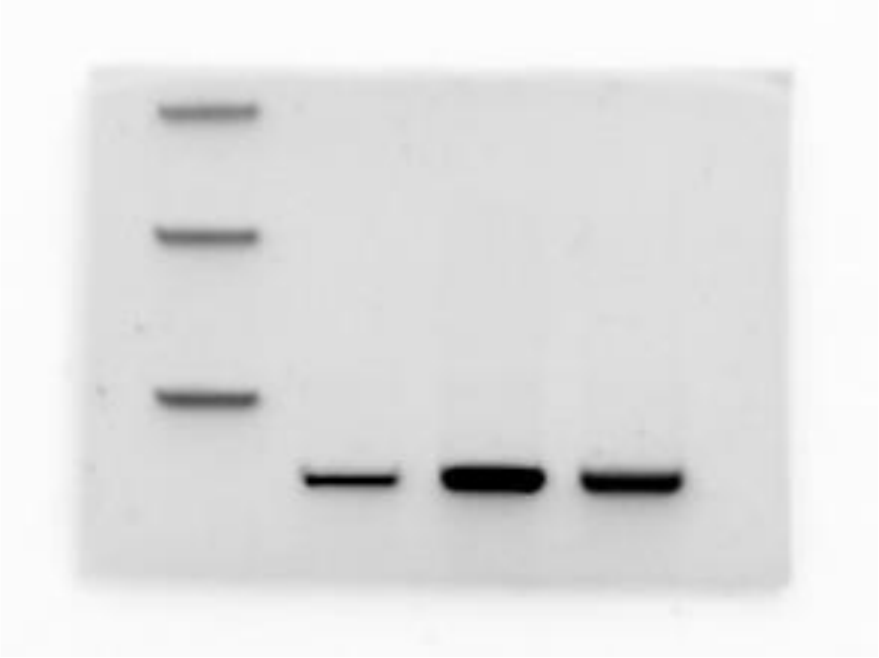

5K-β-actin

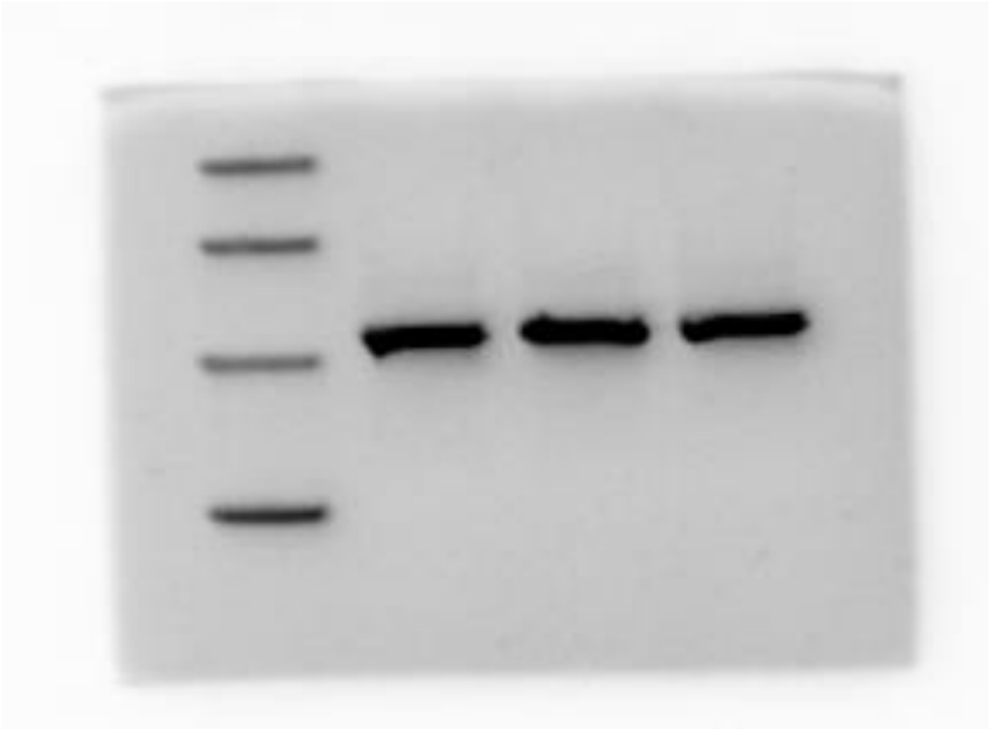

5M-CXCL1
HeLa

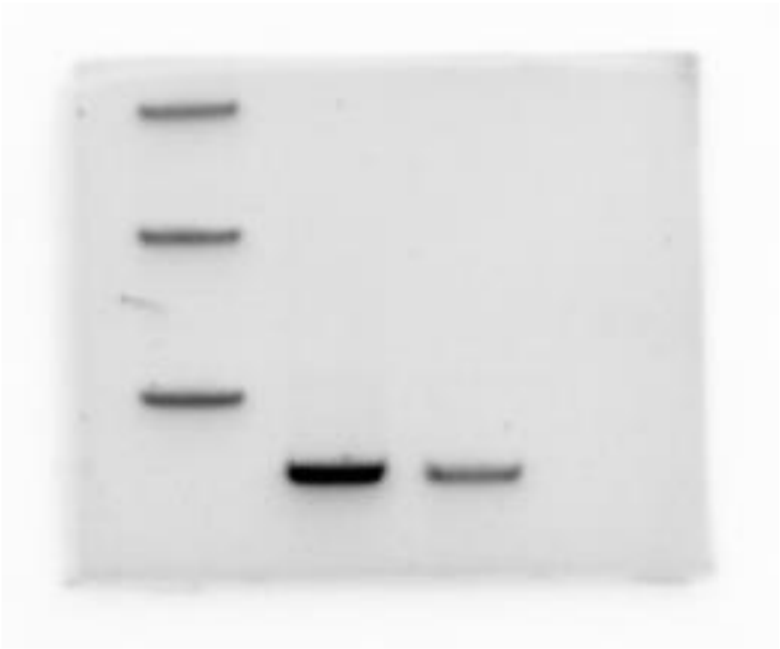

5M-β-actin
HeLa

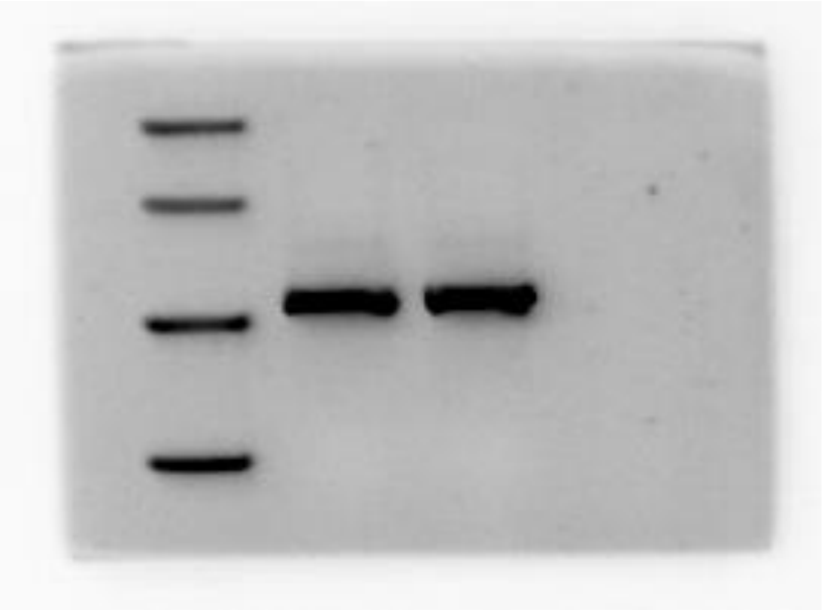

5M-CXCL1
CaSki

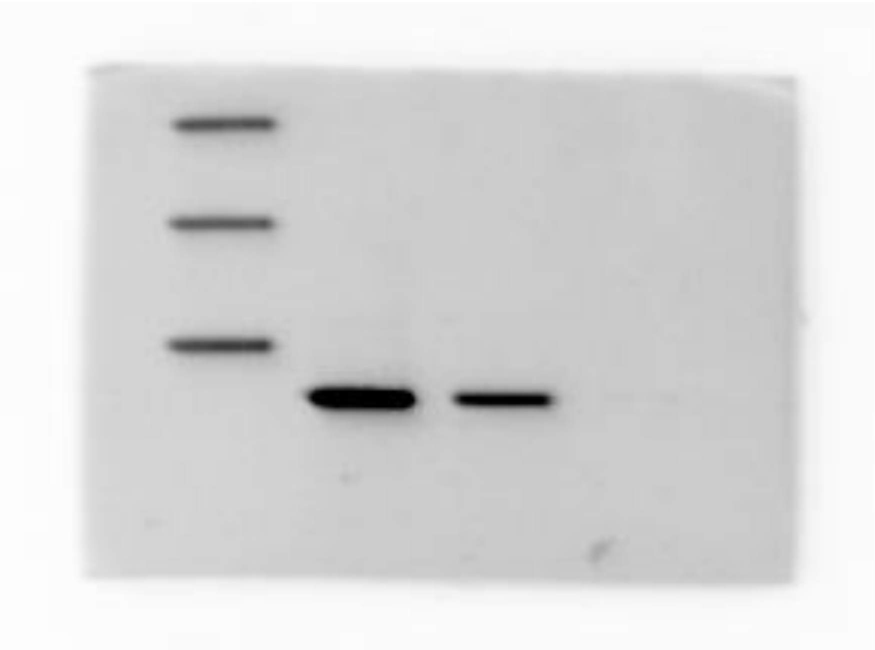

5M-β-actin
CaSki

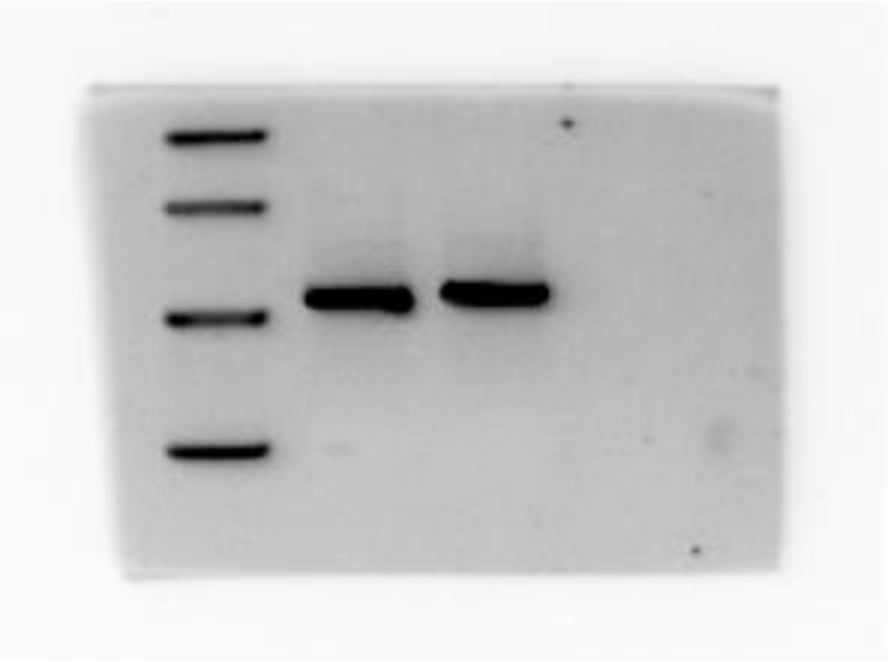

6A-CXCL1
HeLa

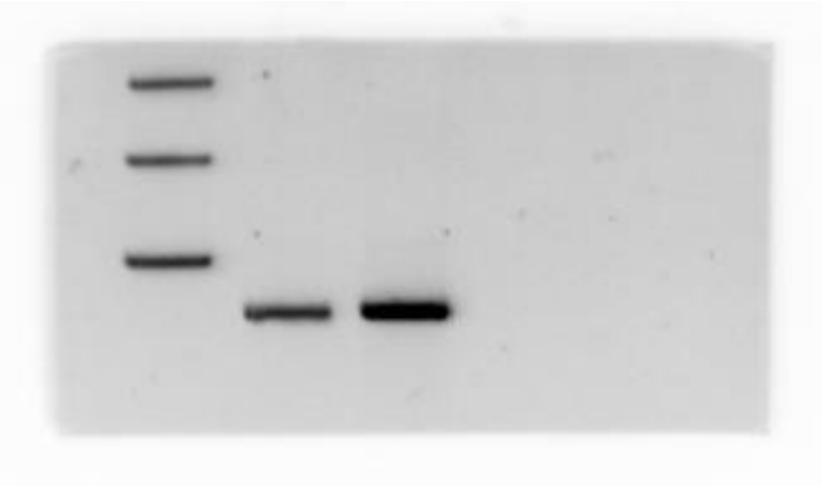

6A-β-actin
HeLa

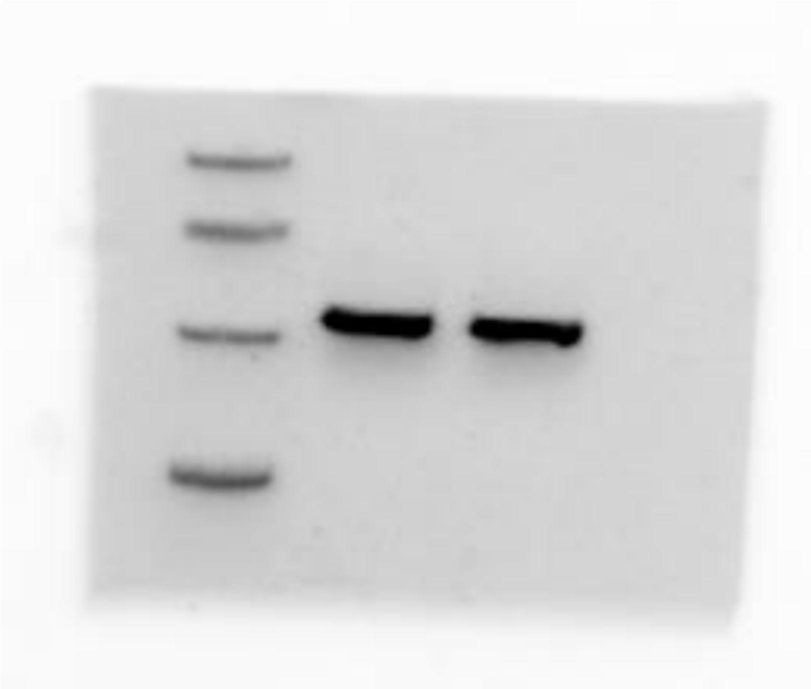

6A-CXCL1
CaSki

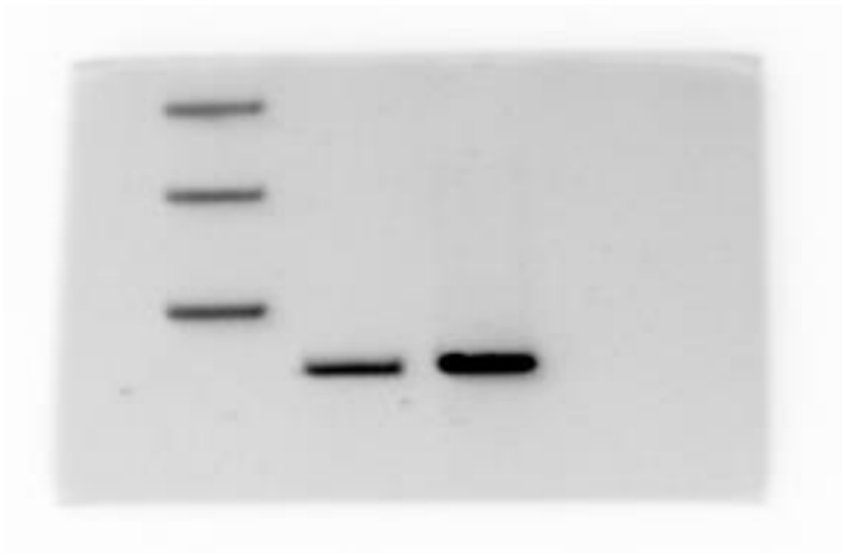

6A-β-actin
CaSki

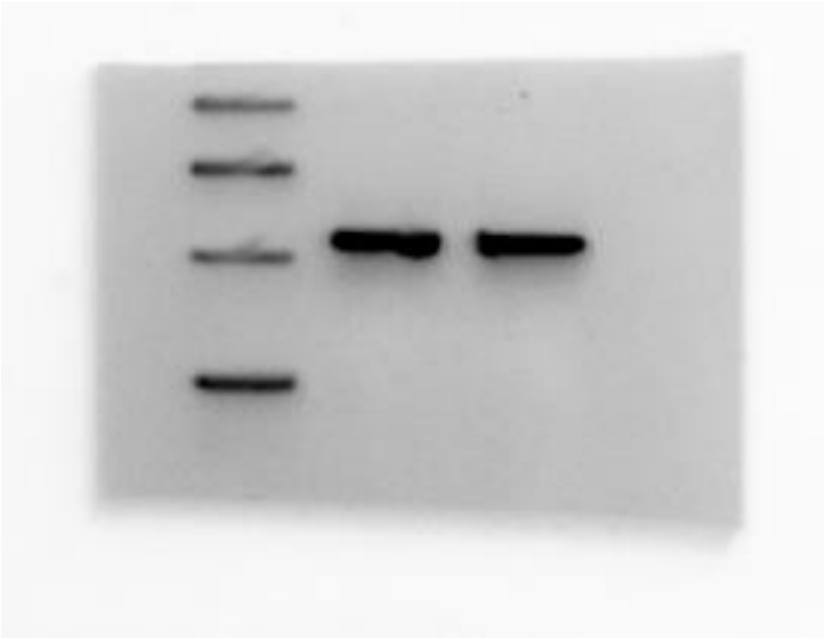

6B-CXCL1
HeLa

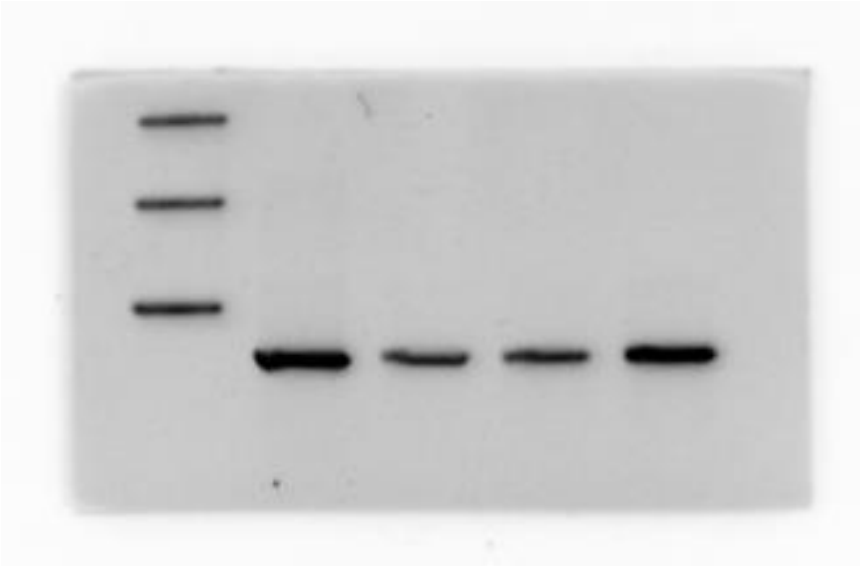

6B-β-actin
HeLa

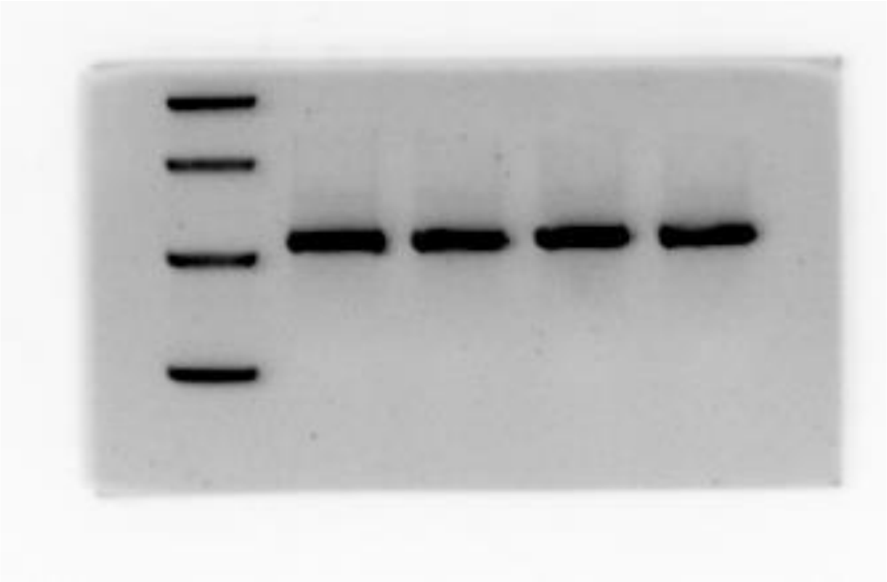

6B-CXCL1
CaSki

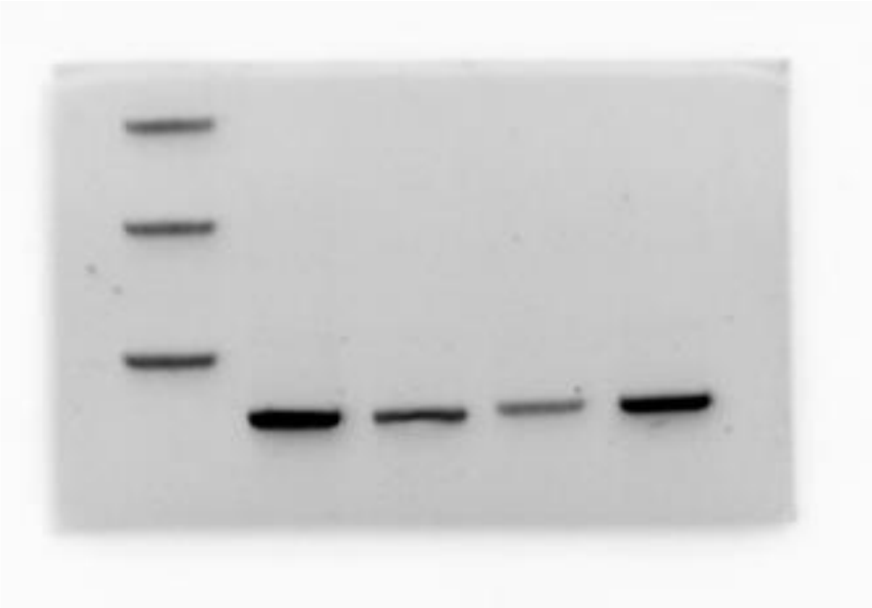

6B-β-actin
CaSki

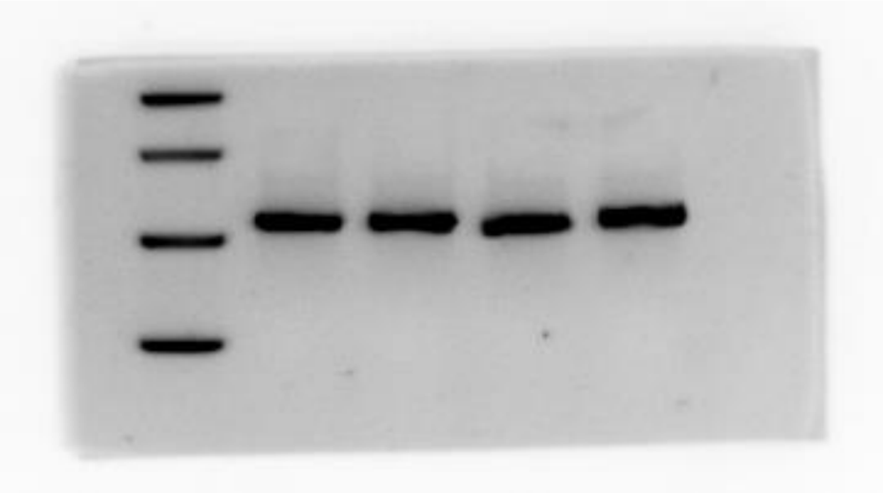

6K-BCL2
HeLa

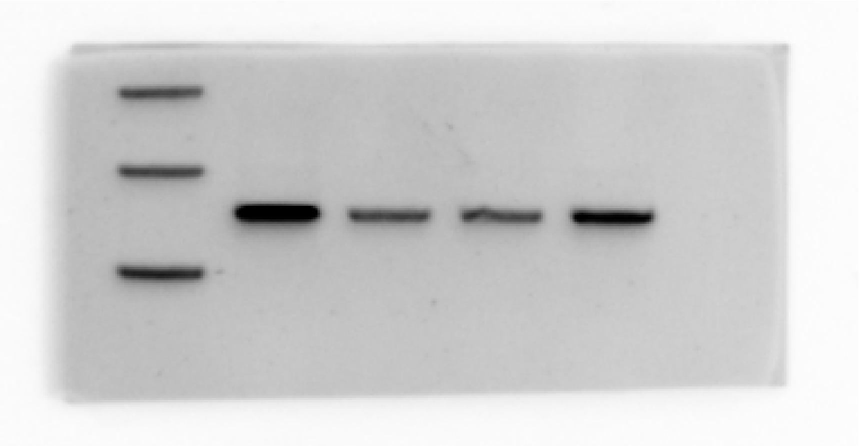

6K-BAX
HeLa

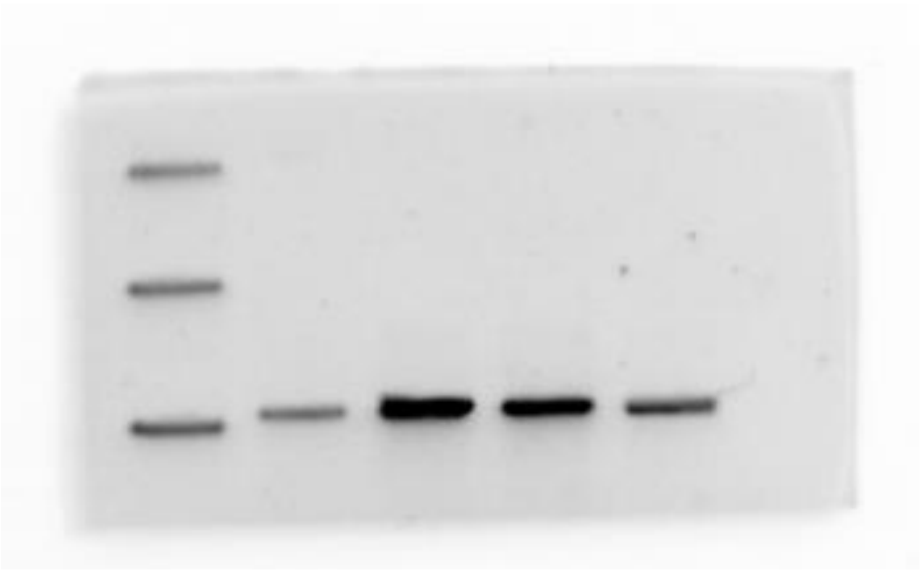

6K-β-actin
HeLa

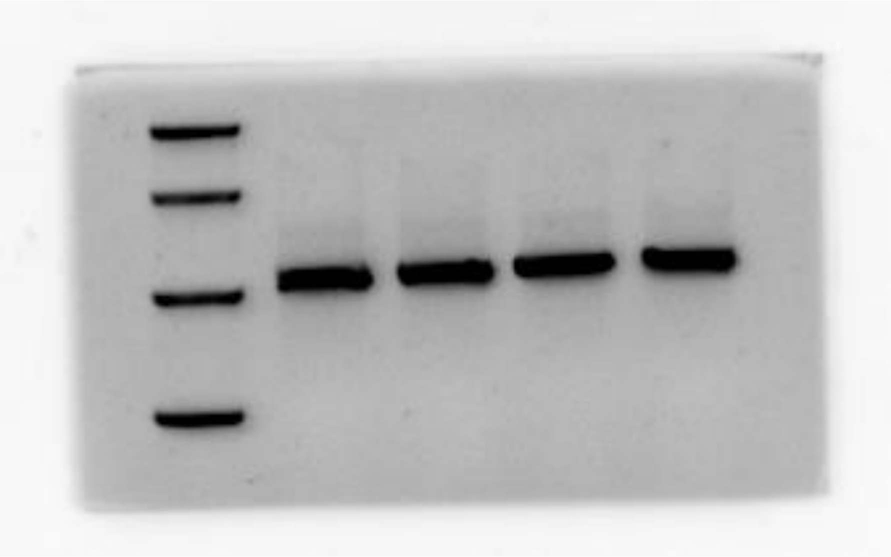

6K-BCL2
CaSki

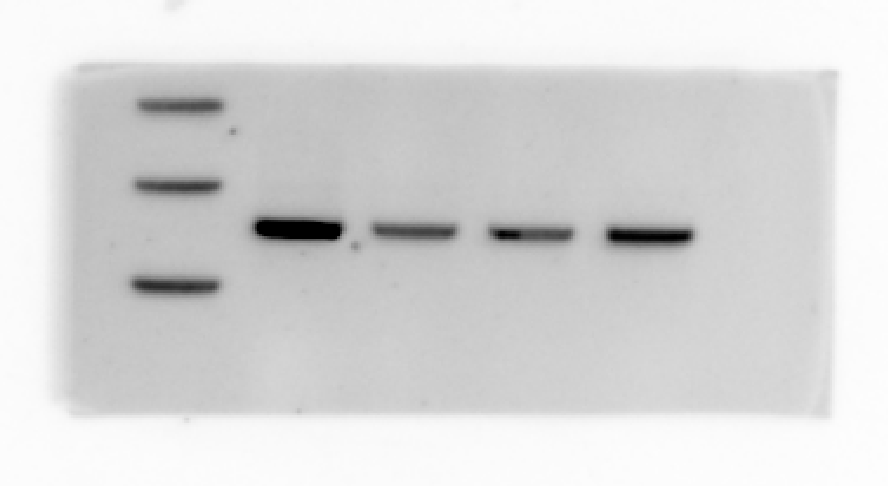

6K-BAX
CaSki

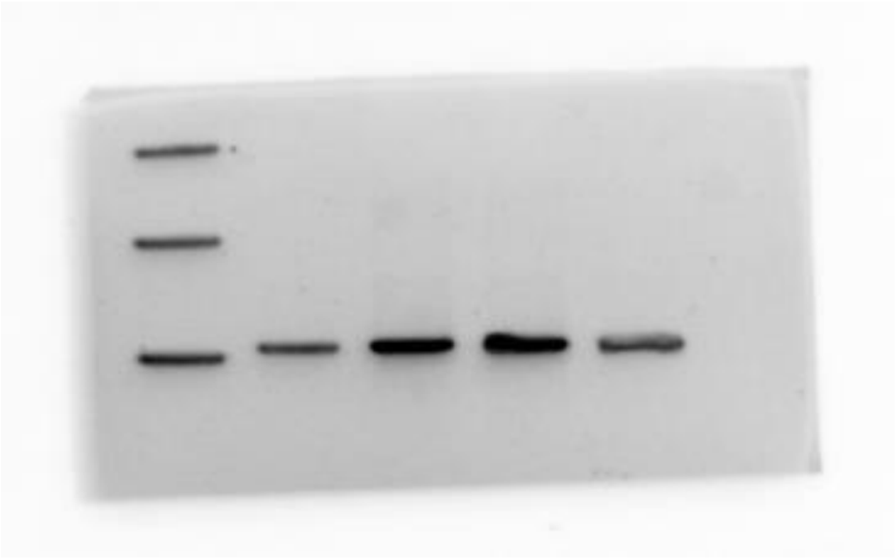

6K-β-actin
CaSki

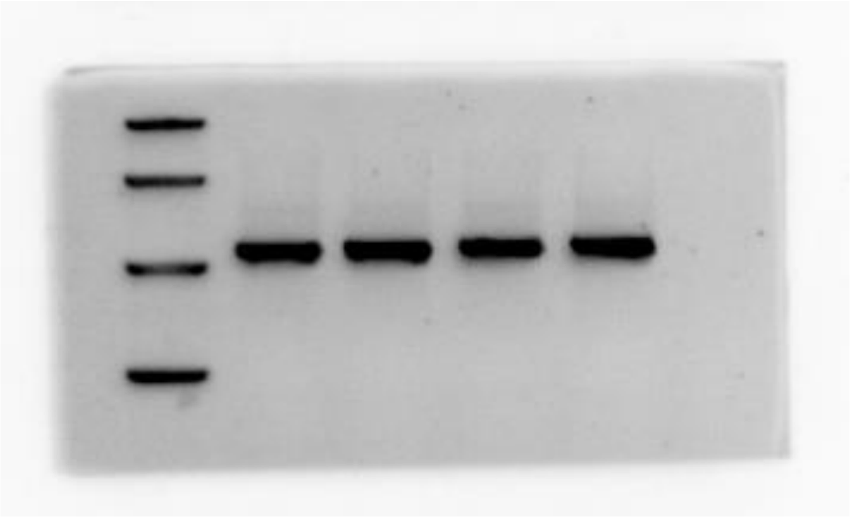

7C-β-actin
HeLa

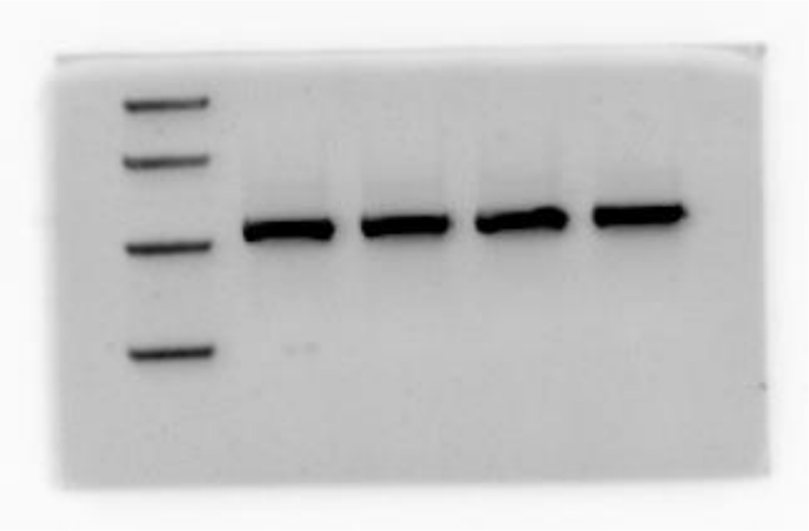

7C-CXCL1
HeLa

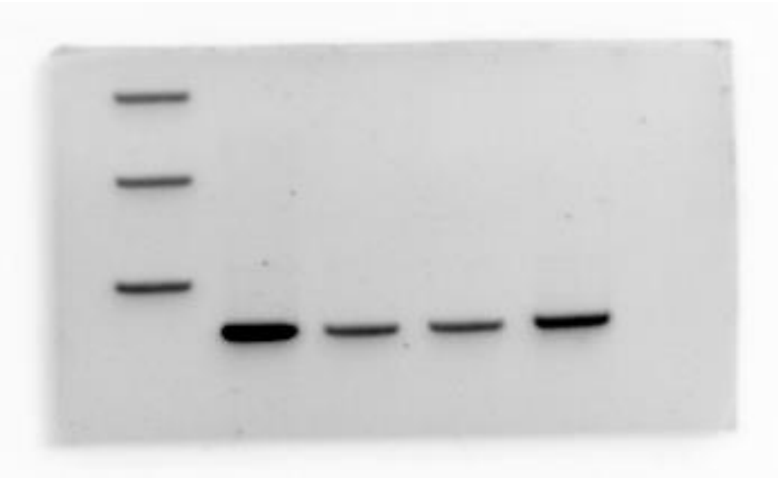

7C-β-actin
CaSki

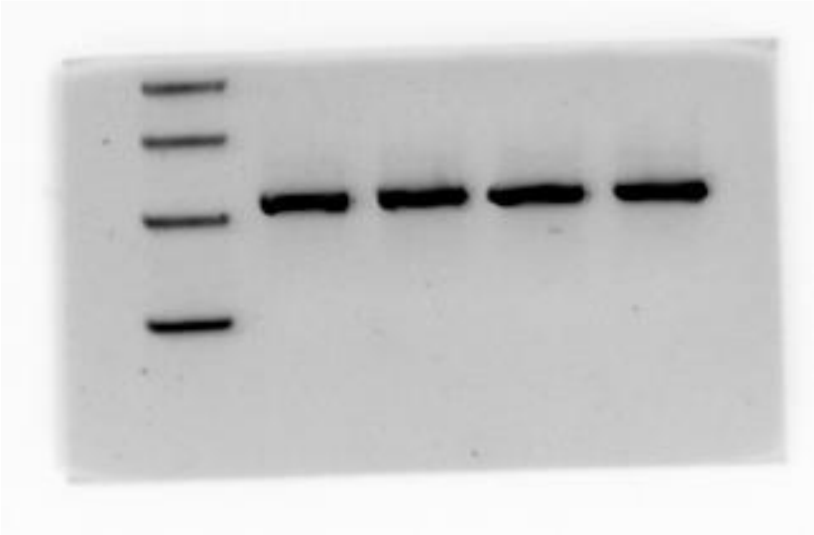

7C-CXCL1
CaSki

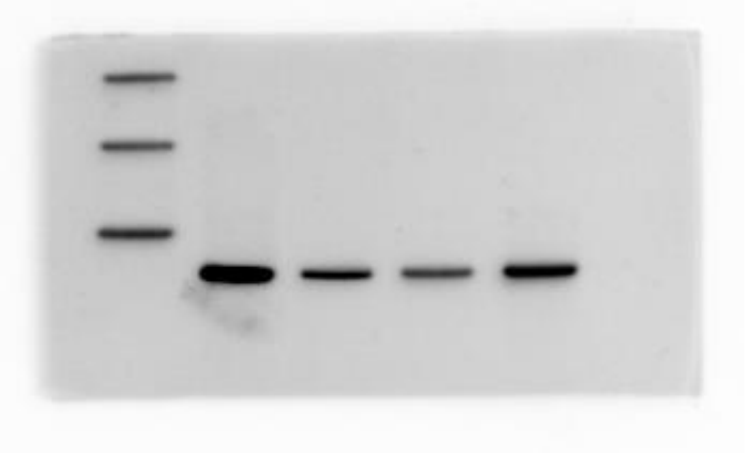

8F-β-actin

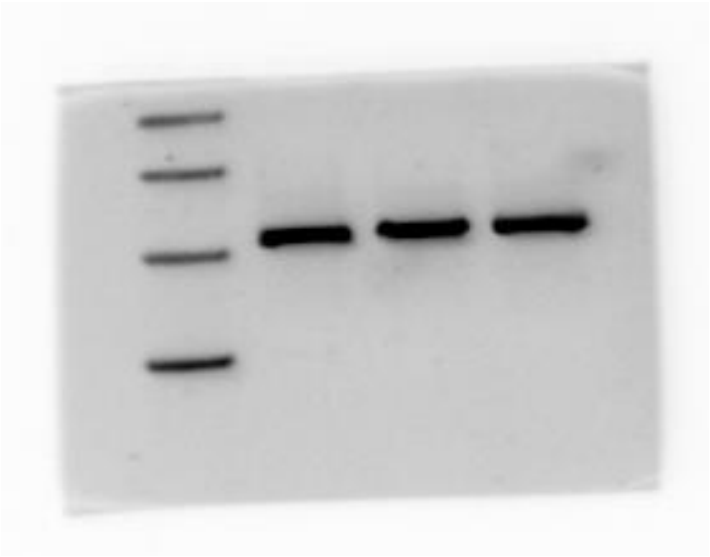

8F-CXCL1

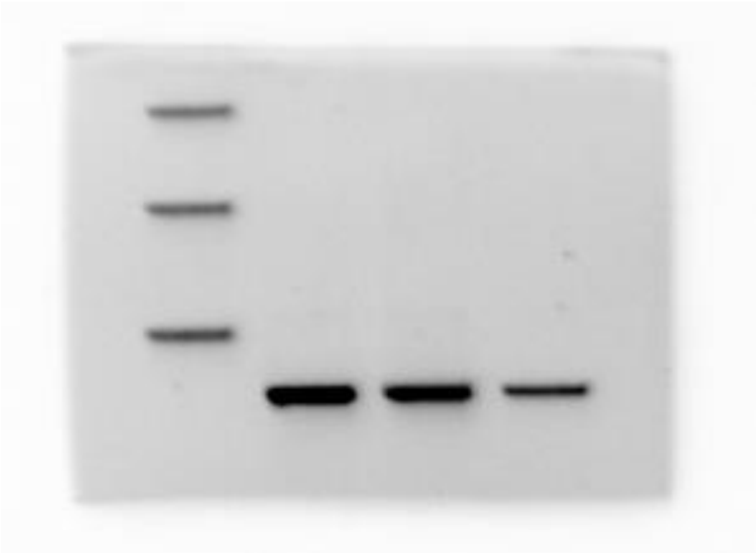

Supplement: Supplementary file 1 — Additional file1: Uncropped images for western blots. [file 12672_2024_933_MOESM1_ESM.pdf]
